# Molecular Signatures of Obesity-Associated Infertility in Polycystic Ovary Syndrome: The Emerging Role of Exosomal microRNAs and Non-Coding RNAs

**DOI:** 10.3390/genes16091101

**Published:** 2025-09-17

**Authors:** Charalampos Voros, Georgios Papadimas, Despoina Mavrogianni, Aristotelis-Marios Koulakmanidis, Diamantis Athanasiou, Kyriakos Bananis, Antonia Athanasiou, Aikaterini Athanasiou, Ioannis Papapanagiotou, Dimitrios Vaitsis, Charalampos Tsimpoukelis, Maria Anastasia Daskalaki, Vasileios Topalis, Marianna Theodora, Nikolaos Thomakos, Fotios Chatzinikolaou, Panagiotis Antsaklis, Dimitrios Loutradis, Evangelos Menenakos, Georgios Daskalakis

**Affiliations:** 1 1st Department of Obstetrics and Gynecology, ‘Alexandra’ General Hospital, National and Kapodistrian University of Athens, 11528 Athens, Greece; depy.mavrogianni@yahoo.com (D.M.); aristoteliskoulak@gmail.com (A.-M.K.); tsimpoukelischa@gmail.com (C.T.); md181341@students.euc.ac.cy (M.A.D.); martheodr@gmail.com (M.T.); thomakir@hotmail.com (N.T.); panosant@gmail.com (P.A.); gdaskalakis@yahoo.com (G.D.); 2 Athens Medical School, National and Kapodistrian University of Athens, 15772 Athens, Greece; dr.georgepapadimas@gmail.com (G.P.); gpapamd@hotmail.com (I.P.); vaitsisdim@gmail.com (D.V.); vtopalismd@gmail.com (V.T.); loutradi@otenet.gr (D.L.); evmenenakos@hotmail.com (E.M.); 3 IVF Athens Reproduction Center V. Athanasiou, 15123 Maroussi, Greece; diamathan16@gmail.com (D.A.); antoathan16@gmail.com (A.A.); diamathan17@gmail.com (A.A.); 4 King’s College Hospitals NHS Foundation Trust, London SE5 9RS, UK; kyriakos.bananis@nhs.net; 5 Laboratory of Forensic Medicine and Toxicology, School of Medicine, Aristotle University of Thessaloniki, 54124 Thessaloniki, Greece; fotischatzin@auth.gr; 6 Fertility Institute-Assisted Reproduction Unit, Paster 15, 11528 Athens, Greece

**Keywords:** polycystic ovary syndrome, PCOS, obesity, infertility, exosomal microRNA, non-coding RNA, biomarker, insulin resistance, folliculogenesis, reproductive endocrinology

## Abstract

Polycystic ovarian syndrome (PCOS) is one of the most common endocrine and metabolic conditions affecting women of reproductive age. This condition affects around 20% of this demographic and is characterized by polycystic ovarian morphology, hyperandrogenism, and chronic anovulation. Obesity, impacting 40–85% of women with PCOS, exacerbates insulin resistance, increases insulin levels, and intensifies low-grade inflammation. This exacerbates the reproductive and metabolic complications associated with the condition. Recent advancements in molecular biology have underscored the significance of non-coding RNAs, including as microRNAs (miRNAs), long non-coding RNAs (lncRNAs), and circular RNAs (circRNAs), as crucial regulators of gene expression and prospective biomarkers for PCOS. Exosome-derived microRNAs (ex-miRNAs) have emerged as compelling candidates due to their stability in body fluids and their capacity to promote intercellular communication among adipose tissue, the ovary, and the endometrium. Research, encompassing both experimental and clinical studies, has shown that ex-miRNAs display differing expression levels in women with obesity-related PCOS. Several of these ex-miRNAs are associated with networks that govern inflammation, glucose metabolism, steroidogenesis, and folliculogenesis. Moreover, the encapsulation of these chemicals within exosomes safeguards them from enzymatic breakdown, hence augmenting their potential as non-invasive biomarkers for diagnosis, prognosis, and treatment monitoring. Despite the initial results being encouraging, challenges remain in standardising exosome separation, quantifying miRNA, and analyzing functional data within the complex pathophysiology of PCOS. This narrative review consolidates existing evidence regarding the molecular signatures of obesity-related infertility in PCOS, emphasising the growing significance of exosomal miRNAs and other non-coding RNAs, while examining their translational potential for early diagnosis and personalised therapeutic approaches.

## 1. Introduction

### 1.1. Molecular Foundations and Pathophysiology

Polycystic ovarian syndrome (PCOS) is a prevalent endocrine–metabolic condition impacting women of reproductive age. The updated 2023 international evidence-based guideline specifies that, after ruling out related conditions, a diagnosis in adults is confirmed if at least two of the following criteria are met: polycystic ovarian morphology (PCOM), clinical and/or biochemical hyperandrogenism, and oligo- or anovulation [1]. Phenotypic classification provides additional clinical insights. The Rotterdam criteria specify four phenotypes: ovulatory dysfunction with polycystic ovarian morphology (phenotype A), hyperandrogenism with ovulatory dysfunction (phenotype B), hyperandrogenism with polycystic ovarian morphology (phenotype C), and ovulatory dysfunction with polycystic ovarian morphology (phenotype D). Phenotype D usually shows milder metabolic symptoms, while phenotypes A–C, typified by hyperandrogenism, are linked with more severe metabolic abnormalities, including insulin resistance and unfavourable cardiometabolic profiles. Genetic susceptibility, epigenetic modulation, and environmental or lifestyle influences merge to precipitate PCOS. These factors result in neuroendocrine and metabolic problems, sustained by self-reinforcing feedback loops marked by insulin resistance, hyperandrogenism, and dysfunction of the hypothalamic–pituitary–ovarian axis. The condition exhibits phenotypic diversity; nonetheless, common unifying traits include hyperandrogenism, chronic anovulation, polycystic ovarian morphology, and a notable prevalence of insulin resistance (IR) [2]. The reproductive and metabolic aspects of PCOS are intricately interconnected. Hyperinsulinemia augments androgen synthesis in theca cells via interactions with insulin and IGF-1 receptors, suppresses the formation of hepatic sex hormone-binding globulin (SHBG), and elevates the bioavailability of androgens. Elevated testosterone levels result in anovulation, impede follicular development, and diminish the aromatase activity of granulosa cells. A harmful loop connecting subfertility to persistent cardiometabolic risk is formed when prolonged anovulation and hyperandrogenism intensify insulin resistance and adipose tissue inflammation.

In PCOS the hypothalamic–pituitary–ovarian (HPO) axis is characterized by persistently elevated gonadotropin-releasing hormone (GnRH) pulsatility, mostly resulting in the secretion of luteinizing hormone (LH) rather than follicle-stimulating hormone (FSH) [3]. The altered LH:FSH ratio induces excessive proliferation of theca cells and overproduction of steroidogenic enzymes such as cytochrome P450c17α (CYP17A1), resulting in increased androgen oogenesis [4]. Hyperandrogenemia impedes granulosa cell aromatase (CYP19A1) activity, thereby interrupting oestradiol production and resulting in follicular arrest during the pre-antral and early antral phases. Anovulation directly results in infertility and is the underlying cause of the characteristic polycystic ovarian shape [5].

Obesity, particularly visceral fat accumulation, acts as both a catalyst and an aggravating factor in the pathophysiology of PCOS. The proliferation of adipose tissue leads to systemic insulin resistance, requiring compensatory hyperinsulinemia [6]. Insulin collaborates with LH on theca cells via the insulin receptor (INSR) and the insulin-like growth factor 1 receptor (IGF1R) to further enhance androgen synthesis [7]. Hyperinsulinemia diminishes the synthesis of hepatic sex hormone-binding globulin (SHBG), resulting in elevated concentrations of free androgens in the bloodstream, hence perpetuating the hyperandrogenic condition [8].

At the molecular level, chronic hyperinsulinemia induces serine/threonine phosphorylation of insulin receptor substrate (IRS) proteins via inflammatory kinases such as c-Jun N-terminal kinase (JNK) and IκB kinase β (IKKβ), thereby impairing downstream phosphatidylinositol 3-kinase/protein kinase B (PI3K/AKT) signalling [9]. This disruption diminishes glucose uptake in skeletal muscle and adipose tissue while enhancing hepatic gluconeogenesis, resulting in a self-sustaining metabolic malfunction that worsens ovarian function.

In obesity, pro-inflammatory M1 macrophages infiltrate adipose tissue, and in conjunction with hypertrophic adipocytes, they secrete cytokines such as tumour necrosis factor-alpha (TNF-α), interleukin-6 (IL-6), and monocyte chemoattractant protein-1 (MCP-1) [10]. These cytokines promote the synthesis of reactive oxygen species (ROS) and activate nuclear factor kappa B (NF-κB) signalling. Oxidative stress in the ovary compromises mitochondrial membrane potential, halts ATP synthesis, and induces granulosa cell apoptosis. Reactive oxygen species (ROS) also alter the signalling of mitogen-activated protein kinase (MAPK) and p38, exacerbating inflammation in the region and inhibiting folliculogenesis [11].

Alongside genetic susceptibility, the pathophysiology of PCOS is affected by epigenetic alterations, including DNA methylation, histone changes, and non-coding RNA-mediated control [12]. Alterations in DNA methylation have been detected in genes associated with insulin signalling (INSR, IRS-1), steroidogenesis (CYP19A1), and gonadotropin response (FSHR). The patterns of histone acetylation and methylation in granulosa cells influence chromatin accessibility at the promoters of critical reproductive genes, thereby impacting their transcriptional activity [12,13].

MicroRNAs (miRNAs), typically 18–24 nucleotides in length, serve as crucial post-transcriptional regulators by binding to complementary sequences in target mRNAs, thereby inhibiting translation or facilitating mRNA degradation [14]. Abnormal miRNA profiles have been identified in ovarian granulosa cells, follicular fluid, serum, and adipose tissue in PCOS [15]. Dysregulated miRNAs modify several signalling pathways essential to PCOS, including the following:

- The PI3K/AKT pathway regulates glucose uptake, utilisation, and survival in granulosa cells.
- The signalling of AMP-activated protein kinase (AMPK) influences cellular energy homeostasis and steroidogenesis.
- The transforming growth factor-beta (TGF-β) pathway regulates follicular development and remodels the extracellular matrix.
- The Wnt/β-catenin signalling pathway affects the proliferation and differentiation of granulosa cells.

Exosomes are extracellular vesicles ranging from 30 to 150 nm in size, secreted via the endosomal pathway. They encompass a diverse array of proteins, lipids, and nucleic acids. In the context of obesity-related PCOS, exosome-derived microRNAs (ex-miRNAs) function as a crucial medium for inter-tissue molecular communication [16]. Exosomes originating from adipose tissue, enriched with particular miRNAs, can affect ovarian granulosa cells, modulating insulin sensitivity, steroidogenic enzyme production, and apoptotic pathways [17].

RNA-binding proteins such as heterogeneous nuclear ribonucleoprotein A2B1 (hnRNPA2B1) and Argonaute 2 (Ago2) facilitate the selective encapsulation of miRNAs into exosomes. This enables the emergence of disease-specific exosomal miRNA signatures [18]. These signals have significant durability in circulation due to the protective lipid bilayer, making them attractive candidates for non-invasive biomarker development. In pPCOS, ex-miRNAs such as miR-93, miR-320a, and miR-103 have been associated with alterations in insulin signalling, granulosa cell proliferation, and androgen synthesis [19,20].

lncRNAs and circRNAs contribute to the molecular pathophysiology of PCOS by functioning as ceRNAs and sequestering miRNAs, hence altering their availability to target mRNAs. Certain circRNAs behave as “miRNA sponges,” influencing pathways associated with ovulatory failure, insulin resistance, and inflammatory responses [21].

The interplay of neuroendocrine dysregulation, metabolic abnormalities, inflammation, oxidative stress, and epigenetic alterations establishes a self-perpetuating pathogenic cycle in obesity-related PCOS. Exosomal non-coding RNAs function as both effectors and biomarkers of this diseased situation, linking systemic metabolic dysfunction to localised ovarian impairment. This mechanistic link strongly supports the investigation of exosomal miRNAs and other non-coding RNAs as potential biological indicators and therapeutic targets for obesity-related infertility in PCOS.

### 1.2. Clinical and Translational Relevance

PCOS is defined by many molecular abnormalities that are not limited to obese persons. Epidemiological studies across many populations demonstrate that PCOS may impact women regardless of their weight, while obesity exacerbates the endocrine-metabolic profile and increases reproductive dysfunction. Consequently, obesity-related PCOS should not be regarded as the only symptom of the condition, despite its more severe clinical appearance [22]. These dysregulations operate at the intersection of endocrine signalling, epigenetic modification, and inflammatory pathways, creating a pathophysiological environment that perpetuates both infertility and systemic disease [23]. A comprehensive understanding of these relationships is crucial for integrating novel molecular biomarkers into precision medicine strategies and for improving personalised therapies in reproductive endocrinology.

Folliculogenesis is negatively impacted by the hyperandrogenic and insulin-resistant environment typical of PCOS, which is common across all phenotypes but commonly worsened in instances of obesity, by disrupting intra-ovarian signalling pathways, including as PI3K/AKT, ERK1/2, and TGF-β/SMAD pathways [24]. Granulosa cells, essential for oocyte development, exhibit transcriptional suppression of genes required for cumulus growth (HAS2, PTGS2, TNFAIP6) and zona pellucida formation, leading to diminished fertilisation capability. Excessive androgens diminish aromatase activity, altering the follicular microenvironment’s equilibrium in favour of increased androstenedione and testosterone levels. This alters the functionality of granulosa cells and accelerates atresia [25].

Insulin resistance aggravates these abnormalities by reducing IGF1 receptor signalling, which is crucial for granulosa cell proliferation and FSH response. The efficiency of oxidative phosphorylation at the mitochondrial level diminishes, partly due to miRNAs reducing the amounts of PGC-1α and nuclear respiratory factors [26]. This results in a reduced availability of ATP for meiotic spindle assembly. Follicular fluid from obese PCOS patients displays unique exosomal miRNA profiles, characterised by an elevation in miR-93, which targets CDKN1A (a regulator of cell cycle arrest), and a reduction in miR-320a, which modulates ESR1 and eNOS [27]. This establishes a direct association between non-coding RNA dysregulation and reduced metaphase II oocyte yield, as well as aberrant embryo cleavage kinetics in ART cycles. These alterations influence endometrial receptivity by modifying integrin levels, Wnt signalling, and local cytokines (such as LIF and IL-6), hence diminishing the likelihood of implantation [28].

Cardiovascular and metabolic risks is increased. Obesity-related PCOS markedly elevates the risk of impaired glucose tolerance, type 2 diabetes mellitus, dyslipidaemia, and hypertension, arising from molecular disruptions that beyond conventional endocrine abnormalities [29]. At the cellular level, continuous activation of stress kinases such as JNK and p38 MAPK in skeletal muscle and adipose tissue impairs IRS phosphorylation, therefore perpetuating insulin resistance. Downregulation of AMPK diminishes fatty acid oxidation and promotes de novo lipogenesis in the liver, exacerbating hepatic steatosis and dyslipidaemia [30].

Circulating exosomal miRNAs both reflect and potentially influence these processes—miR-122 and miR-34a, which are elevated in obese PCOS, facilitate hepatic lipid accumulation by targeting SIRT1 and PPARα, whereas altered levels of vascular-regulatory miRNAs (e.g., miR-320a, miR-126) diminish endothelial nitric oxide synthase activity, thereby reducing nitric oxide bioavailability [31]. This results in endothelial dysfunction, diminished angiogenic capacity, and microvascular rarefaction. In the ovarian environment, compromised microcirculation worsens follicular oxygenation and food supply, creating a bidirectional feedback loop between cardiovascular and reproductive pathologies [32].

Psychiatric comorbidities, such as anxiety and depression, often manifest in PCOS due to psychosocial stressors and direct neuroendocrine–molecular alterations. Body image and sleep disturbances substantially detriment quality of life [33,34]. Dysregulated miRNA expression in hypothalamic neurones, particularly changed miR-132 and miR-124, affects synaptic plasticity and neuropeptide release, thereby affecting GnRH pulsatility and the stress axis activity. This influences the secretion of reproductive hormones and mood control by altering the pathways of CRH and monoaminergic neurotransmitters [33,34].

Chronic low-grade inflammation, characteristic of obesity-related PCOS, compromises the blood–brain barrier through cytokine-induced disruption of tight junctions, enabling peripheral immune mediators to influence hypothalamus and limbic system function [35]. These neuroinflammatory alterations may influence the feedback sensitivity of the HPA axis, thereby perpetuating hypercortisolemia and aggravating gonadotropin secretion dysfunction [36]. The interdependent connection between psychological stress and neuroendocrine dysfunction creates a self-reinforcing cycle that intensifies metabolic and reproductive anomalies due to neuropsychiatric distress [37]. Exosomal miRNAs and other non-coding RNAs hold unique translational importance due to their stability in circulation, encapsulation within lipid bilayer vesicles, and disease-specific expression patterns. Conversely, these molecular signals may be measured frequently with minimal invasiveness and accurately reflect fluctuating physiological conditions [38].

Clinically, they may enhance differential diagnosis by distinguishing between obese PCOS and lean PCOS, as well as other etiologies of anovulation, via molecular fingerprints. Specific miRNA profiles, including the overexpression of miR-21 and the downregulation of miR-132, are associated with oocyte competence, blastocyst formation rates, and live birth outcomes in ART [39]. From a therapeutic monitoring standpoint, changes in circulating exosomal miRNAs may precede measurable hormonal or metabolic shifts, acting as an early signal of treatment success in lifestyle modifications, insulin-sensitizing treatments, or ovulation induction techniques [40].

Non-coding RNAs represent a compelling study domain for novel therapies due to their regulation of critical metabolic, angiogenic, and steroidogenic pathways. The application of antagomiRs to inhibit detrimental miRNAs or synthetic miRNA mimics to restore absent regulatory signals could, theoretically, rectify dysfunctional gene networks in PCOS [41]. Delivery by modified exosomes offers tissue specificity, allowing for the targeted delivery to granulosa cells or endometrial stromal cells, hence reducing systemic off-target effects. Furthermore, the modification of exosomal miRNA content using pharmaceutical agents—such as metformin, GLP-1 receptor agonists, or selective androgen receptor modulators—may indirectly restore reproductive and metabolic equilibrium. These techniques are currently in the preclinical phase, aligning with the broader movement towards precision reproductive endocrinology, which integrates molecular diagnostics with tailored treatments [6,42].

To fully exploit the clinical potential of these biomarkers, standardisation of techniques is important. Standardisation of procedures for exosome isolation (ultracentrifugation versus size-exclusion chromatography), RNA extraction, and normalisation of quantitative PCR or sequencing data is essential for enabling comparability and reproducibility of investigations.

Integrating exosomal non-coding RNA profiling into diagnostic algorithms may improve PCOS phenotyping, particularly in obese patients where metabolic and reproductive disorders converge. This approach may facilitate the development of personalised ART protocols that enable targeted interventions, such as tailored gonadotropin dose or metabolic optimisation prior to ART, to enhance reproductive results and long-term metabolic health.

## 2. PCOS, Obesity, and Infertility: A Pathophysiological Synopsis

Obesity constitutes a significant risk factor for PCOS. It exacerbates reproductive and metabolic issues by a combination of endocrine, inflammatory, and molecular mechanisms. In the context of PCOS, high adiposity exacerbates insulin resistance and hyperandrogenism while substantially altering the ovarian microenvironment, hence impeding folliculogenesis, oocyte quality, and endometrial receptivity. This section analyses the molecular and physiological mechanisms both confirmed and speculative that connect obesity to the reproductive and metabolic characteristics of PCOS, highlighting the interplay among adipose tissue-derived factors, the hypothalamic-pituitary-ovarian axis, and molecular mediators such as microRNAs and exosomes.

The Rotterdam criteria, requiring at least two indicators—oligo- or anovulation, clinical and/or biochemical hyperandrogenism, and PCOM—after ruling out related disorders, constituted the principal framework for diagnosing PCOS in the examined studies. WHO defined obesity as a BMI of 30 kg/m^2^ or above, whereas a BMI ranging from 25 to 29.9 kg/m^2^ is classified as overweight. “Obese anovulation” is notably different from PCOS. Anovulation in obese women without hyperandrogenism or polycystic ovarian morphology should be categorised as a predominantly metabolic phenotype instead of PCOS. To guarantee precise interpretation of the data, we have meticulously maintained this difference throughout the text, distinguishing between obesity-related anovulation without PCOS and obese PCOS.

### 2.1. Adipose Tissue as an Endocrine and Paracrine Organ in PCOS

In PCOS, particularly in the presence of obesity, adipose tissue functions as a metabolically active organ that interacts with the immune system, capable of influencing systemic metabolism and the local ovarian microenvironment through endocrine, paracrine, and autocrine signalling processes [43]. Obesity is marked by adipocyte hypertrophy and hyperplasia, accompanied by ECM remodelling, typified by increased deposition of fibrotic components such as collagen VI, which restricts tissue expandability and promotes cellular hypoxia [44]. Hypoxic stress has been shown to stabilise HIF-1α, hence augmenting the production of pro-angiogenic proteins such as VEGF and a range of inflammatory mediators, including TNF-α, IL-1β, and MCP-1. MCP-1 promotes the recruitment of pro-inflammatory M1 macrophages, altering the immunological equilibrium in adipose tissue to a cytokine-rich catabolic state [45].

TNF-α and IL-6 stimulate NF-κB and JAK/STAT pathways in adipocytes and immune cells, establishing a self-perpetuating inflammatory loop that disseminates low-grade inflammation systemically and alters ovarian stroma functionality [46]. These cytokines penetrate the ovarian blood barrier, attaching to their particular receptors on granulosa and theca cells. This interaction stimulates NF-κB, therefore enhancing the synthesis of steroidogenic enzymes such as CYP17A1 and STAR. This enhances androgen production and results in the hyperandrogenic phenotype characteristic of PCOS [47].

Adipose tissue serves as a secretory hub for adipokines that directly influence reproduction. Leptin, elevated in individuals with more adiposity, activates JAK2/STAT3 and PI3K/AKT signalling pathways in ovarian cells [48]. Excessive leptin over an extended period can induce cellular resistance, hence impeding the proliferation of granulosa cells and the synthesis of steroids. Conversely, adiponectin, typically diminished in individuals with excess weight, exhibits anti-inflammatory properties and enhances insulin sensitivity through the activation of AMPK. The reduced adiponectin levels in obese PCOS patients further predispose them to insulin resistance and follicular dysfunction [49].

An developing aspect is the function of adipose-derived extracellular vesicles—particularly exosomes—as transmitters of molecular signals to the ovary. These vesicles encompass miRNAs, lncRNAs, and proteins that alter critical pathways in the ovaries. For example, miR-27a and miR-155 present in adipose-derived exosomes might diminish IRS1 levels and alter PI3K/AKT signalling, rendering granulosa cells less susceptible to gonadotropin stimulation. Additional exosomal cargo, such as heat shock proteins (HSP70, HSP90), can activate toll-like receptor 4 (TLR4)-dependent inflammatory pathways in ovarian cells, exacerbating oxidative stress and cellular apoptosis [50].

Lipolytic activity in hypertrophic adipocytes leads to elevated circulation FFAs, which are taken up by ovarian cells via CD36 and FATP transporters. Excessive accumulation of FFA leads to lipotoxicity, mitochondrial dysfunction, and ER stress. This stimulates UPR sensors such as PERK and IRE1α [51]. Endoplasmic reticulum stress can impede steroid hormone synthesis by modifying the expression and localisation of enzymes like aromatase and 3β-HSD, resulting in diminished oestradiol production. Molecular communication between defective adipose tissue and the ovaries in obese women with PCOS is mediated by a complex network of cytokines, adipokines, lipids, and nucleic acids produced from extracellular vesicles. This systemic-local axis not only perpetuates metabolic dysregulation but also directly impairs folliculogenesis, oocyte maturation, and the capacity for successful implantation [23,52].

### 2.2. Insulin Resistance, Hyperinsulinemia, and Ovarian Dysfunction

In PCOS, IR arises from a multifaceted impairment of post-receptor insulin signalling, encompassing deficits in INSR autophosphorylation, exposure of IRS1/2 docking sites, and activation of downstream kinases [53]. JNK, IKKβ, mTOR/S6K1, and PKCθ can excessively phosphorylate IRS proteins with serine residues, hence inhibiting normal tyrosine phosphorylation. This functions as a biochemical “brake” on the transmission of insulin signals [54]. The alteration in phosphorylation inhibits the p85 PI3K subunit from associating with IRS, hence decelerating the transformation of PIP2 to PIP3 and complicating the phosphorylation of AKT at both Thr308 and Ser473. In GC, the reduction in AKT activity inhibits the phosphorylation of AS160, hence preventing GLUT4 vesicles from translocating to the plasma membrane [55]. The reduction in glucose input diminishes glycolytic intermediates, limits pyruvate transfer across CX43/CX37 gap junctions to the oocyte, and obstructs ATP and NADPH availability for meiotic advancement [56].

This metabolic deficit is worsened by altered nuclear–cytoplasmic dynamics of FOXO1. When AKT activity is diminished, FOXO1 remains in the nucleus and inhibits the transcription of genes such as G6PD and PRDX3, which are crucial for maintaining redox homeostasis [57]. Decreased NADPH synthesis hinders glutathione regeneration, making GC vulnerable to ROS-induced DNA strand breakage and lipid peroxidation. Concurrently, FOXO1 promotes the transcription of pro-apoptotic mediators such as Bim, FasL, and TRAIL, hence shifting the equilibrium towards GC apoptosis and early follicular atresia. The resultant effect is a follicular milieu that is less capable of facilitating high-quality oocyte maturation, hence impacting embryo viability [58].

HI introduces an additional dimension of dysfunction by directly altering TC steroidogenesis. Insulin amplifies LH signalling by co-activating the MAPK/ERK and PI3K/AKT pathways, hence accelerating cholesterol import into mitochondria via STAR and augmenting the transcription of CYP11A1, CYP17A1, and HSD3B2 [59]. This hyperactivation results in excessive androgen synthesis, which subsequently inhibits GC aromatase (CYP19A1) by disrupting the FSHR–cAMP–PKA axis. Reduced E2 synthesis modifies the androgen/estrogen ratio within the follicle, serving as a biochemical indicator of follicular arrest in PCOS [60]. HI diminishes the synthesis of hepatic SHBG via inhibiting HNF-4α. This elevates the concentrations of free testosterone in the bloodstream, hence accentuating HA-related traits [61].

The interplay between metabolic and inflammatory signalling markedly intensifies this dysfunction. HI interacts with adipokines such as TNF-α, IL-6, resistin, and leptin to activate NF-κB and STAT3 in ovarian and peripheral tissues [62]. The transcription of SOCS3, induced by NF-κB, leads to the degradation of IRS1/2 via the proteasome, exacerbating insulin resistance. Concurrently, FFA from hypertrophic adipocytes interacts with TLR4 on GC and TC, initiating MyD88-dependent cascades that activate p38 MAPK and JNK [63]. This results in the prolonged secretion of cytokines (IL-1β, MCP-1) and the activation of the inflammasome (NLRP3). This chronic low-grade inflammation exacerbates insulin and gonadotropin signalling, perpetuating the ovary’s metabolic and hormonal maladaptation [64].

This nutrient-rich and inflammatory milieu will consistently induce stress in the mitochondria. Under high-intensity conditions, elevated substrate flow excessively drives electron transport chain complexes I and III, resulting in enhanced electron leakage and superoxide production [65]. The accumulation of ROS damages mtDNA, accelerates the oxidation of cardiolipin, and activates PARP1, depleting NAD+ reserves and inhibiting SIRT1 activity. The suppression of the SIRT1–PGC-1α pathway obstructs mitochondrial biogenesis and undermines antioxidant defences [66]. The altered fission-fusion equilibrium, resulting from DRP1 hyperphosphorylation and MFN2 downregulation, compromises the integrity of the cristae, hence diminishing the efficacy of oxidative phosphorylation and ATP generation. A deficiency in GC energy results in insufficient substrate for the oocyte, complicating spindle formation and chromosomal segregation [67].

HI alters the molecular composition of GC-derived extracellular vesicles in follicular fluid, hence modifying the functionality of reproductive signals. Elevated insulin levels alter exosomal miRNA profiles by increasing miR-29a, miR-320, and miR-483, while decreasing miR-222 levels [68]. These miRNAs influence critical insulin signalling genes (INSR, PIK3R1, AKT2) and steroidogenic regulators (STAR, CYP19A1), hence propagating insulin resistance and steroidogenic dysregulation to adjacent granulosa cells [69]. Exosomal miRNAs can alter the pathways associated with CC expansion genes (HAS2, PTGS2) and oocyte meiotic development (CDC25B, WEE2), hence complicating fertility [70].

From a therapeutic perspective, therapies designed to restore PI3K/AKT activity and diminish serine phosphorylation of IRS proteins are essential for breaking this cycle. MET activates AMPK, which inhibits mTOR/S6K1, reduces IRS serine phosphorylation, and accelerates GLUT4 translocation. Incorporating MYO–DCI into the diet enhances PIP3 synthesis, hence activating AKT and translocating FOXO1 to the cytoplasm. Both techniques have been shown to partially normalise FF exosomal miRNA profiles, improve E2/T ratios, and augment MII oocyte production in obese PCOS phenotypes receiving ART. The twin benefits of metabolic correction and reproductive improvement underscore the translational importance of concentrating on the insulin–gonadotropin–miRNA axis in this scenario.

### 2.3. Chronic Low-Grade Inflammation and OS in PCOS

PCOS presents as a chronic, systemic, low-grade inflammatory disorder arising from the interplay of metabolic anomalies, hormonal abnormalities, and immunological dysfunction. In adipose tissue, especially visceral fat, hypertrophic adipocytes experience hypoxic stress due to inadequate angiogenesis, which activates HIF-1α and enhances the transcription of VEGF, GLUT1, and several pro-inflammatory cytokines, including as TNF-α, IL-6, and MCP-1 [71]. These mediators regulate the recruitment of monocytes and their differentiation into M1-polarized macrophages. The macrophages subsequently secrete IL-1β, IL-18, and more TNF-α, which activates NF-κB and AP-1, so intensifying the inflammatory cycle [72]. This cascade affects metabolic balance by promoting SOCS3 expression, which binds to IRS1/2 and directs them for proteasomal destruction, hence disrupting PI3K-AKT insulin signalling. This cascade affects metabolic balance by promoting SOCS3 expression, which binds to IRS1/2 and directs them for proteasomal destruction, hence disrupting PI3K-AKT insulin signalling [73]. Concurrently, insulin-resistant adipocytes secrete increased quantities of free fatty acids into the bloodstream, which interact with TLR4 on macrophages and ovarian cells. This initiates the MyD88-dependent recruitment of IRAK4 and TRAF6, resulting in the phosphorylation of IKKβ and the translocation of NF-κB p65/p50 heterodimers into the nucleus [74]. This promotes the transcription of pro-inflammatory genes and activates the NLRP3 inflammasome, facilitating the cleavage of pro-caspase-1 into active caspase-1, which subsequently converts pro-IL-1β into mature IL-1β. The systemic cytokine load affects peripheral insulin sensitivity and directly regulates ovarian steroidogenic and follicular cells in a paracrine fashion [75].

The inflammatory microenvironment of the ovary interacts with local immune cell activity to modify follicular function. TC subjected to TNF-α and IL-6 demonstrate hyperactivation of the MAPK/ERK and p38 pathways, leading to the overexpression of STAR, CYP17A1, and HSD3B2, which subsequently amplifies androgen production [76]. Concurrently, GC display compromised FSHR signalling due to cytokine-induced suppression of Gαs–adenylyl cyclase interaction, leading to decreased cAMP levels and reduced PKA-mediated phosphorylation of CREB, which in turn diminishes the transcription of CYP19A1 and aromatase activity, thereby limiting E2 production [77]. The steroidogenic profile biassed towards androgens hinders the maturation of oocytes within the follicular niche. Furthermore, persistent cytokine exposure amplifies the expression of NOX2 and NOX4, resulting in elevated ROS generation that adversely impacts mitochondrial complexes I and III, hence exacerbating oxidative phosphorylation. This disturbance results in the buildup of NADH and electron leakage, generating superoxide anions that convert into H_2_O_2_ and, in the presence of transition metals, into hydroxyl radicals via Fenton chemistry. This induces lipid peroxidation, protein oxidation, and DNA strand breakage [78,79].

At the mitochondrial level, oxidative damage induced by ROS serves as both a cause and a consequence of PCOS disease. Excessive ROS depolarises the mitochondrial membrane potential (ΔΨm) in germ cells, resulting in the opening of the mitochondrial permeability transition pore (mPTP) and the release of cytochrome c into the cytosol [80]. This interacts with Apaf-1 to create the apoptosome, which then activates caspase-9 and ultimately caspase-3, resulting in apoptosis. This cellular demise diminishes the concentrations of metabolites such as pyruvate, amino acids, and lipids essential for oocyte competence [81]. Furthermore, ROS inflict damage on mtDNA by inducing oxidative lesions, particularly within the D-loop region. This complicates the transcription of ETC subunits such as ND1, ND4, and COX1, perpetuating mitochondrial dysfunction [82]. Lipid peroxidation products, notably 4-HNE and MDA, form Michael adducts with cysteine, lysine, and histidine residues on spindle-associated proteins, such as tubulins and kinesins. This diminishes the reliability of the spindle assembly checkpoint and increases the likelihood of aneuploidy. The oxidative carbonylation of actin filaments in GC also impairs the cytoskeletal dynamics essential for cumulus expansion and oocyte release [83].

Inflammatory signalling alters the cargo composition of ovarian EVs. Upon exposure to cytokines and ROS stress, GC and T cells secrete EVs abundant in pro-inflammatory microRNAs such as miR-155, miR-146a, and miR-21 [27]. These miRNAs inhibit target mRNAs associated with insulin signalling (INSR, AKT2), steroidogenesis (CYP19A1, HSD17B1), and mitochondrial antioxidant defences (SOD2, GPX4). These EVs also contain DAMPs such as HMGB1 and S100A8/A9, which interact with RAGE and TLR4 on the recipient cells. This enhances NF-κB signalling and cytokine secretion. This vesicle-mediated intercellular communication transmits the inflammatory signal within the follicular niche and potentially across the system, so linking ovarian dysfunction to systemic metabolic impairment. Extracellular vesicles derived from the follicular fluid of patients with polycystic ovary syndrome exhibit alterations in lipid raft composition, characterised by an increased presence of ceramides and sphingomyelins. This can alter the clustering of membrane receptors and the downstream signalling of cells, introducing an additional layer of molecular regulation to the inflammatory network [84,85].

The molecular connection between oxidative stress and inflammation is centred on the redox-sensitive transcription factor NF-κB. Reactive oxygen species oxidise thiol groups on cysteine residues of protein tyrosine phosphatases such as PTEN and PP2A, hence prolonging NF-κB activation and kinase phosphorylation [86]. This results in the continuous transcription of IL-1β, IL-6, TNF-α, and adhesion molecules such as ICAM-1 and VCAM-1, hence perpetuating leukocyte recruitment. Oxidative alteration of Keap1, conversely, liberates Nrf2, which promotes the transcription of ARE-regulated antioxidant genes such as SOD1/2, GPX4, and HO-1 [87]. In PCOS, prolonged inflammation diminishes Nrf2 nuclear translocation via GSK-3β-mediated phosphorylation, undermining antioxidant defences and promoting oxidative dominance. This mismatch increases the likelihood of GC undergoing ferroptosis, characterised by iron-dependent lipid peroxidation and compromised membrane integrity. Ferroptotic GC released oxidised phosphatidylethanolamines and extracellular vesicles loaded with pro-ferroptotic miRNAs, resulting in follicular attrition [88].

Therapeutic strategies targeting the inflammation–oxidative stress axis in PCOS include MET, which activates AMPK, inhibits NF-κB p65 phosphorylation at Ser536, and prevents NLRP3 inflammasome formation by downregulating TXNIP. Inositol supplementation has been shown to affect PI3K-AKT signalling, reduce circulating levels of TNF-α and IL-6, and improve insulin sensitivity. Antioxidants such as resveratrol and NAC restore ΔΨm, replenish GSH levels, and reduce lipid peroxidation in GC, hence enhancing oocyte competence. Evidence indicates that these medicines can reinstate the normal extracellular vesicle miRNA profile, hence reducing pro-inflammatory cargo and enhancing intercellular communication within the follicular milieu. Future therapy approaches may focus on dual-targeting techniques that simultaneously modulate NF-κB and Nrf2 pathways, aiming to restore redox balance and alleviate chronic inflammation, thereby addressing the underlying molecular pathology of PCOS.

### 2.4. Hyperandrogenism and Ovarian Microenvironment Remodeling

Hyperandrogenism in PCOS denotes a multifaceted endocrine condition that profoundly impacts ovarian function. It must be distinguished from non-PCOS types of hyperandrogenism, such as adrenal hyperplasia, androgen-secreting neoplasms, or drug-induced androgen excess, which possess unique pathophysiological origins and clinical trajectories. In PCOS, hyperandrogenism arises from the interaction of ovarian theca cell dysfunction, insulin resistance, and altered hypothalamic-pituitary signalling, resulting in a chronic and systemic reproductive-metabolic imbalance. This arises from an aberrant interplay between LH hypersecretion, HI, and dysregulated intraovarian signalling, leading to sustained activation of TC steroidogenesis [89]. Elevated concentrations of LH activate the LHCGR–Gαs–adenylyl cyclase–cAMP signalling cascade. This facilitates PKA’s phosphorylation of transcription factors such as CREB, while also interacting with MAPK/ERK and PI3K-AKT signalling pathways to enhance the synthesis of steroidogenic enzymes [90]. The enhancement of STAR-mediated cholesterol import into the inner mitochondrial membrane facilitates the conversion of cholesterol into pregnenolone by CYP11A1. Concurrently, the activities of CYP17A1 and HSD3B2 increase, resulting in excessive production of A4 and T. These androgens migrate to GC, where defective FSHR–cAMP–PKA signalling inhibits CYP19A1 from converting androgens into E2 [91]. This results in the FF being saturated with androgens. This androgen-dominant environment alters the transcriptome and proteome of germ cells, cumulus cells, and the oocyte. It also alters the expression of ECM remodelling enzymes (MMP2, MMP9, TIMP1), gap junction proteins (CX43, CX37), and angiogenic regulators such as VEGFA. This cascade disrupts cumulus expansion, intercellular metabolic coupling, and follicular vascularization, thus impeding follicular maturation and ovulatory potential [92].

Besides its steroidogenic actions, HA directly impacts the bioenergetics and oxidative stress of GC mitochondria. Upon activation of AR in these cells, it initiates the transcription of genes critical for mitochondrial biogenesis (PGC-1α, TFAM) and lipid β-oxidation (CPT1A, ACADVL) [93]. While this may theoretically enhance oxidative metabolism, in the context of PCOS, it is linked to substrate overload, an excessive influx of NADH/FADH_2_ to the ETC, and subsequent electron leakage, leading to elevated production of mitochondrial ROS. [94]. Reactive oxygen species oxidize cardiolipin, compromising the integrity of the mitochondrial membrane and impairing the functionality of complexes I and III. Simultaneously, AR signalling alters mitochondrial motility, promoting DRP1-mediated fission while inhibiting MFN1/2- and OPA1-dependent fusion. This increases the likelihood of mitochondrial network depolarization [95]. This impedes ATP production, leading to an insufficient energy supply for meiotic spindle formation, chromosomal alignment, and cytokinesis during egg maturation. Oxidative damage triggers mitochondrial apoptotic pathways through BAX/BAK oligomerization, cytochrome c release, and activation of caspase-9/3. This reduces the quantity of functional GCs required to sustain the oocyte [96].

Epigenetically, HA orchestrates substantial chromatin remodeling in ovarian somatic cells. Upon ligand binding to the AR, it undergoes dimerization and translocates to the nucleus, where it associates with androgen response elements (AREs) in the promoters of steroidogenic and metabolic genes [97]. AR recruits co-activators like as p300/CBP, which possess intrinsic histone acetyltransferase activity, to enhance H3K27ac and transcriptional activity. In alternative genomic settings, it recruits HDACs or the NuRD complex to establish a repressive chromatin configuration [98]. The epigenetic modifications induced by AR influence steroidogenesis, oxidative defence (SOD2, GPX4), and insulin sensitivity (IRS1, INSR). HA alters the production and activity of DNMT1, DNMT3A, and DNMT3B, resulting in the hypermethylation of CpG islands in genes such as CYP19A1 and the hypomethylation of AR target genes. This exacerbates detrimental transcriptional pathways affecting health. These alterations may persist regardless of androgen levels, indicating the presence of a permanent “epigenetic memory” that maintains malfunction throughout reproductive cycles [99].

The inflammatory effects of HA stem from reciprocal interactions between AR and inflammatory signalling networks. AR activation enhances the transcriptional activity of NF-κB via recruiting p300 to acetylate p65, prolonging p65’s nuclear retention, and promoting phosphorylation via IKKβ. This increases the likelihood of transcription of IL-6, TNF-α, MCP-1, and CCL20, perpetuating the inflammatory cycle within the follicle [100]. ROS generated by androgen-enhanced NOX activity serve as secondary messengers, activating redox-sensitive kinases (ASK1, JNK) that subsequently amplify NF-κB and AP-1 activity, resulting in increased cytokine production. Concurrently, HA induces the synthesis of TXNIP, a recognised activator of the NLRP3 inflammasome [101]. This recruits ASC, activates caspase-1, and facilitates the maturation of IL-1β and IL-18. Upon activation of the inflammasome, it induces pyroptosis in GC, resulting in the release of DAMPs such as HMGB1 and mitochondrial DNA into the FF. This subsequently triggers TLR9 and RAGE signalling in adjacent cells. This exacerbates inflammation, inhibits steroidogenesis, and compromises the structural integrity of the follicle [102].

Hyaluronic acid substantially modifies extracellular vesicle-mediated intercellular communication in the ovary. Germ cells and testicular cells under androgenic stress secrete extracellular vesicles abundant in microRNAs, including miR-21, miR-125b, miR-222, and miR-93 [103]. These miRNAs target genes associated with insulin signalling (IRS1, PIK3R1), cellular proliferation (CDKN1A, PTEN), and steroidogenic regulation (CYP19A1, STAR). A proteome analysis of HA-derived extracellular vesicles reveals an increased presence of AR protein, HSP90, lipid metabolism enzymes (ACLY, FASN), and inflammatory mediators (S100A8/A9), which can alter the phenotypic of recipient cells [104]. Alterations in EV lipids encompass elevated concentrations of ceramides and sphingomyelins, which affect membrane fluidity, lipid raft stability, and receptor clustering. This impacts signalling pathways such as PI3K-AKT and MAPK. The ingestion of these extracellular vesicles in the oocyte may alter mitochondrial function, spindle assembly, and transcriptome readiness for fertilisation, hence reinforcing the association between hyaluronic acid and reduced developmental competence [105].

From a translational perspective, addressing HA requires comprehensive techniques aimed at adjusting the androgen–estrogen ratio and restoring follicular homeostasis. The use of COC to reduce LH levels diminishes TC androgen secretion, while MET enhances insulin sensitivity, hence indirectly decreasing ovarian androgen synthesis. Anti-androgens like spironolactone and flutamide obstruct AR activation, therefore mitigating subsequent transcriptional, mitochondrial, and inflammatory changes. Experimental techniques include AR co-regulator inhibitors, NOX antagonists to reduce oxidative stress, and therapies designed to normalise EV cargo via metabolic and hormonal control. Epigenetic treatments, such as HDAC inhibitors and DNMT modulators, has the capacity to reverse HA-induced chromatin configurations, albeit they remain in the preclinical stage. Restoring aromatase activity—either by direct pharmacological enhancement or by mitigating androgen-induced suppression—remains a vital treatment goal to create a milieu conducive to maximum oocyte competence and IVF success.

## 3. Extracellular Vesicle-Mediated Mechanisms in Obese PCOS

### 3.1. EV Biogenesis and Secretion Pathways in the PCOS Microenvironment

The biogenesis of EVs in the ovarian microenvironment of obese individuals with PCOS is a consequence of chronic metabolic dysregulation, hormonal excess, and sustained inflammatory signalling, each influencing the vesicle production machinery at distinct cellular checkpoints [106]. The endosomal origin route begins with the invagination of the plasma membrane, leading to the development of early endosomes, a process primarily regulated by clathrin-mediated and caveolin-mediated endocytosis. In PCOS, sustained hyperinsulinemia results in the hyperactivation of PI3K–AKT, which enhances the recruitment of Rab5-GTP to early endosomes and accelerates their maturation [107]. Subsequently, these early endosomes transition to late endosomes, where the generation of ILVs is regulated by the standard ESCRT complexes (0–III) and auxiliary proteins such as CHMP4B and VPS36. NF-κB elevates the concentrations of several auxiliary molecules during inflammation [108]. The ubiquitination state of transmembrane proteins dictates their preferential integration into ILVs via the ubiquitin-binding domains of ESCRT-0 subunits (e.g., HRS, STAM1). This pathway is modified in PCOS, promoting the incorporation of proteins associated with androgen production (e.g., CYP17A1) and insulin signalling regulators [109]. Furthermore, ceramide-mediated ESCRT-independent budding requires increased activity of neutral sphingomyelinase 2 (nSMase2), whose expression is elevated by JNK activation—a process initiated by free fatty acid-induced TLR4 signalling in obese settings. This alters the lipid composition of MVB membranes, resulting in an increased presence of sphingolipids. This reduces the energy barriers for membrane curvature and accelerates ILV budding [110].

Conversely, microvesicle formation occurs directly at the plasma membrane via outward budding. In PCOS, this mechanism is excessively driven by a distinctive interplay of cytoskeletal dynamics, lipid asymmetry regulation, and intracellular calcium signalling. Elevated free fatty acids and hyperglycemia augment DAG concentrations, hence activating conventional PKC isoforms that phosphorylate MARCKS (myristoylated alanine-rich C-kinase substrate) [111]. This induces a conformational alteration in actin filaments. The concurrent activation of the RhoA–ROCK–LIMK–cofilin pathways induces actin polymerisation and alters cortical tension, hence shaping vesicles [112]. On the lipid front, enhancing the activity of aminophospholipid translocase (flippase) and activating scramblase—both induced by Ca^2+^-dependent TMEM16F—facilitates the translocation of phosphatidylserine to the extracellular space. This not only facilitates membrane curvature but also transmits a “eat-me” or “recognize-me” signal to target cells. The elevated intracellular Ca^2+^ required for these processes in PCOS is maintained by impaired store-operated calcium entry (SOCE), where oxidative stress–induced disulphide crosslinking amplifies STIM1 and Orai1 channel interactions, enabling persistent Ca^2+^ influx that drives membrane blebbing cycles [113,114].

Intracellular stress mechanisms that alter vesicle trafficking fidelity increase the rate of extracellular vesicle production in obese PCOS. Mitochondrial ROS precede MAPK p38α and ERK1/2 by phosphorylating SNAP23 and syntaxin-4, so facilitating the adhesion and fusion of vesicles with the plasma membrane [115]. Concurrently, hypoxia in expanded adipose tissue and inadequately vascularised ovarian stroma stabilises HIF-1α, which interacts with hypoxia response elements in the promoters of Rab GTPases such as Rab27a, Rab27b, and Rab35. These are crucial for regulating MVB tethering and secretion [116]. Furthermore, endoplasmic reticulum stress via the IRE1α–XBP1s pathway of the UPR facilitates the synthesis of membrane lipids essential for extracellular vesicle formation and concurrently enhances Sec22b-dependent trafficking from the endoplasmic reticulum to the Golgi apparatus, thereby aiding the incorporation of secretory proteins into vesicles [117]. Insulin resistance markedly impairs AMPK activity, obstructing autophagosome–lysosome fusion and diverting MVBs from degradation to exocytic release, hence increasing the accumulation of extracellular vesicles.

The hormonal microenvironment in PCOS adds an additional layer of specificity to the generation of EVs. Activation of AR in granulosa and theca cells enhances the transcription of syntenin-1, a scaffold protein essential for ALIX-mediated cargo loading into ILVs. This modifies the vesicular contents to incorporate pro-androgenic miRNAs (such as miR-21 and miR-93) and enzymes associated with steroidogenesis. Simultaneously, hyperinsulinemia-induced decrease in FOXO1 removes the transcriptional inhibition on genes that facilitate the organisation of vesicle lipid rafts, such as flotillin-1 and caveolin-1. These genes alter the composition of membrane microdomains, facilitating the clustering of signalling complexes within budding vesicles. In obesity, elevated levels of TNF-α and IL-6 indicate low-grade chronic inflammation. These levels induce post-translational modifications to ESCRT components via simoylation, which further refines cargo selection and the velocity of vesicle release. Collectively, these molecular alterations provide a hypersecretory extracellular vesicle phenotype characterised by distinct molecular signatures that illustrate the interplay between metabolic and endocrine systems in the aetiology of PCOS. This pattern preserves local paracrine dysregulation in the ovary while simultaneously conveying endocrine-like signals to distant metabolic organs, hence sustaining systemic insulin resistance, inflammation, and reproductive dysfunction.

### 3.2. Molecular Cargo Alterations: miRNAs, lncRNAs, and Proteins

The molecular cargo within EVs in obese PCOS demonstrates a highly selective and regulated sorting process, profoundly affected by the metabolic, hormonal, and inflammatory states of the source cells. Within the category of short non-coding RNAs, miRNAs represent the most extensively researched subset [118]. The altered expression patterns in PCOS-derived EVs indicate their significant function in modifying gene expression networks within local ovarian compartments and remote metabolic organs. The biogenesis and selective loading of miRNAs into extracellular vesicles require the identification of EXO-motifs in miRNA sequences by RNA-binding proteins, including hnRNPA2B1, YBX1, and SYNCRIP, which are subject to post-translational modifications like sumoylation and methylation, affected by hyperinsulinemia and chronic inflammation [119]. In obese PCOS, the extracellular vesicle miRNA profile is skewed towards pro-inflammatory and metabolic-disruptive species, including miR-21, miR-146a, miR-93, miR-103, and miR-122 [120]. Mechanistically, miR-93 targets IRS-1 and CDKN1A, leading to the suppression of insulin signalling pathways and the disruption of cell cycle control in granulosa cells. miR-103 impedes insulin-mediated glucose absorption by downregulating CAV1, hence obstructing caveolae-dependent activation of the insulin receptor [121]. Conversely, miR-122, frequently increased in hepatic EVs, regulates lipid biosynthesis pathways by inhibiting SREBP1 and ACC1, hence influencing systemic dyslipidaemia. Additionally, inflammatory cytokines such as TNF-α and IL-6 activate NF-κB and STAT3 signalling pathways, which alter miRNA transcription and bias Argonaute 2–dependent loading towards species that intensify oxidative stress, mitochondrial dysfunction, and androgen biosynthesis, thereby creating a self-perpetuating pathogenic cycle [122].

Besides miRNAs, extracellular vesicles in obese PCOS are rich in lncRNAs that serve as crucial transcriptional and post-transcriptional regulators. The targeted accumulation of lncRNAs, including H19, MALAT1, and PVT1, within vesicles is enabled by nuclear RNA export mechanisms, specifically NXF1 and CRM1, in conjunction with RNA–protein interactions involving HuR (ELAVL1) and IGF2BP1/2, which are elevated in hyperandrogenic and insulin-resistant conditions [123]. Upon delivery to recipient cells, these lncRNAs function as ceRNAs, sequestering miRNAs that target genes critical for steroidogenesis, cellular proliferation, and extracellular matrix remodelling. For example, H19 sponges let-7 family miRNAs, resulting in the de-repression of IGF2. This enhances PI3K–AKT–mTOR activation in theca cells and elevates testosterone synthesis via CYP17A1 [124]. MALAT1 modulates the alternative splicing of pre-mRNAs encoding meiotic regulators such as MOS and CDC25B, hence influencing the timing of oocyte maturation. PVT1 stabilises oncogenic transcription factors such as c-Myc in granulosa cells, resulting in excessive proliferation while impairing appropriate cellular differentiation. Concurrently, EV-associated lncRNAs delivered to adipocytes and hepatocytes alter metabolic gene expression by affecting chromatin remodelling complexes, such as SWI/SNF and PRC2, consequently linking ovarian dysfunction, systemic insulin resistance, and hepatic steatosis [125].

The proteome composition of EVs in obese polycystic PCOS is complex, comprising enzymes, receptors, structural proteins, and signalling intermediates that collectively modify the metabolic and reproductive environment [126]. Steroidogenic enzymes such as CYP11A1, HSD3B2, and CYP19A1 are incorporated into extracellular vesicles derived from theca and granulosa cells. This enables cells to exchange their capacity to produce androgens and oestrogens with one another [127]. Endometrial stromal cells can internalise these vesicles, potentially altering the equilibrium of oestrogen and progesterone in the region, which may influence the receptivity of implantation. EVs contain numerous inflammatory mediators, including HMGB1, which interacts with TLR4 in macrophages, initiating MyD88-dependent NF-κB activation, and S100A8/A9, which enhances ROS production and activates the NLRP3 inflammasome [128]. The altered expression of adhesion molecules such as integrin αvβ3 and tetraspanins CD9/CD63/CD81 affects tissue tropism and absorption efficiency, demonstrating a significant predilection for endometrial and hepatic tissues in obese PCOS animals. Regarding metabolism, proteins like as SOCS3 and PTP1B are specifically incorporated into vesicles by the ubiquitin–ESCRT machinery regulated by TSG101 and ALIX [129,130]. This inhibits insulin and leptin receptor signalling in skeletal muscle and adipose tissue. These EV-mediated protein transfers sustain systemic insulin resistance and intensify the intraovarian hyperandrogenic state. The presence of heat shock proteins (e.g., HSP70, HSP90) in PCOS extracellular vesicles functions as molecular chaperones that stabilise signalling complexes in recipient cells, hence exacerbating the aberrant activation of the MAPK and PI3K–AKT pathways [106].

The altered EV cargo in obese PCOS serves as a sophisticated communication network in which miRNAs regulate post-transcriptional processes, lncRNAs influence transcriptional and epigenetic pathways, and proteins directly modify receptor-mediated signalling and enzymatic activities. This convergence of molecular processes creates a feedback system linking adipose tissue, the ovary, skeletal muscle, liver, and endometrium, thus sustaining both metabolic dysregulation and reproductive failure. Disrupting specific nodes in this network, such as interactions between miRNA and mRNA, lncRNA-mediated ceRNA circuits, or EV–receptor binding events, may effectively halt the disease cycle of obese PCOS.

### 3.3. Pathophysiological Impact of EV Cargo on Ovarian and Metabolic Tissues

The pathophysiological effects of EV cargo in obese PCOS are determined by its ability to cause significant transcriptome, proteomic, and metabolic reprogramming in endocrine and metabolic organs, leading to a chronic pro-inflammatory and hyperandrogenic state [131]. In the ovarian compartment, EVs released by hyperandrogenic theca cells, insulin-resistant granulosa cells, apoptotic luteal cells, and inflamed ovarian stromal fibroblasts function as effective paracrine and autocrine signalling agents, transmitting molecular directives that interfere with follicular recruitment, selection, and ovulation [132]. MicroRNAs prevalent in extracellular vesicles from patients with polycystic ovary syndrome—particularly miR-21, miR-93, miR-146a, and miR-483—are selectively encapsulated via ceramide-dependent, ESCRT-independent mechanisms and internalised by granulosa and cumulus cells through clathrin-coated pits, caveolae-mediated endocytosis, and micropinocytosis. This ensures that the payload reaches the perinuclear region, where Argonaute2-associated RISC complexes facilitate post-transcriptional silencing [133]. These miRNAs directly suppress genes related to cumulus expansion (HAS2, PTGS2, TNFAIP6) and aromatase expression (CYP19A1), diverting the FSHR–cAMP–PKA–CREB signalling cascade from oestrogen production to androgen predominance. Concurrently, lncRNAs like as H19 and MALAT1, present in EVs, function as competitive endogenous RNAs (ceRNAs), sequestering miRNAs that typically inhibit IGF2 and CCND1. This excessively activates the IGF2–IGF1R–PI3K–AKT and Cyclin D1–CDK4/6 pathways, resulting in granulosa cell proliferation without enough luteinisation. Protein cargo, including HMGB1, S100A8/A9, annexins, and heat shock proteins, associates with TLR4 and RAGE on granulosa cells. This initiates the MyD88–IRAK–TRAF6–NF-κB and p38 MAPK pathways, resulting in elevated production of IL-1β, IL-6, and TNF-α in the region [134]. These proteins induce oxidative stress by generating ROS from NOX4. The disparity between antioxidant defences (SOD, GPx, catalase) and ROS production accelerates mitochondrial depolarisation, cytochrome c release, and caspase-3 activation. This exacerbates the difficulty for oocyte mitochondria to synthesise ATP and prevents the spindle from disintegrating.

EV signalling enables a reciprocal interaction between reproductive and metabolic pathways in adipose tissue. This crosstalk links the reactions of ovarian and peripheral organs to molecular signals from adipocytes, resulting in dysfunction across the whole endocrine system. Insulin-resistant hypertrophic adipocytes secrete EVs that contain miR-27a, miR-34a, miR-122, and SOCS3 [106]. Theca and granulosa cells internalise these extracellular vesicles by integrin αVβ3-mediated docking, subsequently transporting them to endosomes. Upon entry, these cargo components IRS1/2, PI3K regulatory subunits (p85α), and the translocation of AKT–AS160–GLUT4, exacerbating ovarian insulin resistance [135]. This localised anomaly interacts with hyperinsulinemia to enhance ovarian CYP17A1 activity by stimulating the ERK1/2–SF1 pathway independently of PI3K. This elevates androgen production and diminishes SHBG synthesis in the liver, hence augmenting bioavailable testosterone levels. Conversely, EVs secreted by the ovary in PCOS encompass miR-103, PTP1B, and pro-inflammatory proteins that affect adipocytes, thereby enhancing IKKβ–NF-κB signalling, phosphorylating IRS-1 at Ser307, and facilitating the degradation of insulin receptor β-subunits via proteasomes, exacerbating insulin sensitivity [136]. Metabolic crosstalk induced by EVs also influences hepatocytes. The uptake of vesicles containing miR-103 and PTP1B inhibits insulin’s suppression of gluconeogenic genes (G6PC, PCK1) by retaining FoxO1 in the nucleus. This induces fasting hyperglycemia, increased VLDL secretion, and alterations in lipid distribution that exacerbate metabolic stress [106].

In skeletal muscle, extracellular vesicles from women with PCOS induce substantial alterations in mitochondrial function, energy substrate utilisation, and insulin sensitivity, resulting in a systemic metabolic dysregulation. Extracellular vesicle-associated microRNAs, including miR-29a, miR-155, and miR-499, directly target transcriptional coactivators such as PGC-1α and NRF1/2, thereby reducing mitochondrial biogenesis and impeding the expression of electron transport chain complexes (NDUFB8, COX4I1, ATP5A) [137]. The resultant reduction in oxidative phosphorylation capacity results in an increased reliance on anaerobic glycolysis, lactate buildup, and the activation AMPK stress pathways [138]. Simultaneously, protein cargo such as integrin αvβ3 and fibronectin fragments within EVs interact with muscle cell receptors, activating FAK–Src–p38 MAPK signalling. This enhances the transcription of IL-6, CCL2, and TNF-α by NF-κB, establishing a persistent low-grade inflammatory milieu that impedes insulin’s ability to facilitate glucose uptake [139]. EVs also influence the endometrium. For instance, miR-210 and miR-543 in ovarian extracellular vesicles influence the HIF–VEGF and Wnt/β-catenin pathways, respectively. This impedes the ability of blood vessels to alter their morphology, hinders stromal cells from undergoing decidualisation, and obstructs the functionality of receptivity markers such as LIF and integrin αVβ3. These alterations impede the communication between the embryo and the endometrium, hence reducing the likelihood of pregnancy for obese PCOS patients receiving ART [140].

Besides metabolic and reproductive tissues, EV cargo significantly modifies immune system dynamics, intensifying systemic inflammation and tissue infiltration. Monocytes and macrophages exposed to PCOS-derived extracellular vesicles enriched with HMGB1, S100 proteins, and miR-155 undergo NLRP3 inflammasome priming and activation, mediated by mitochondrial reactive oxygen species and potassium efflux through P2X7 receptor channels [141]. This process culminates in the active secretion of IL-1β and IL-18, which disrupt ovarian hormone regulation by inhibiting aromatase and accelerating androgen production in theca cells. Concurrently, endothelial cells internalise EV, resulting in a reduction in eNOS levels via miR-34a and miR-126 [142]. This reduces blood availability and complicates the delivery of blood to the ovaries and uterus. The hypoperfused microenvironment elevates ROS levels further, stabilises HIF1α, and promotes the synthesis of angiogenesis-inhibiting factors such as thrombospondin-1 [143]. The release of MMP-2 and MMP-9 through extracellular vesicles disturbs the extracellular matrix, promoting immune cell infiltration and perpetuating tissue remodelling processes that are adverse in the reproductive context. The alterations in the endothelium and immune system establish a self-sustaining inflammatory-vascular feedback loop, illustrating the systemic impact of PCOS in individuals with obesity [144].

### 3.4. EV Uptake Mechanisms and Intracellular Signaling

The internalisation of EVs in the pathophysiological context of obese PCOS is dictated by a complex interplay among vesicle surface molecules, ECM components, and recipient cell membrane receptors, all meticulously regulated by the systemic metabolic and hormonal milieu [106]. Recent proteomic investigations indicate that absorption facilitated by the syndecan–syntenin–ALIX complex is crucial in follicular granulosa cells. In these cells, heparan sulphate chains of syndecans interact with EV-enriched heparin-binding growth factors, therefore activating Rac1–WAVE–Arp2/3 and altering the actin cytoskeleton [145]. Furthermore, lectin-glycan recognition systems, particularly galectin-3 and galectin-9, facilitate the docking of extracellular vesicles in hyperglycaemic and hyperinsulinemic microenvironments, which are prevalent in PCOS [146]. In these situations, atypical glycosylation patterns on EV surfaces increase the likelihood of binding. Lipid raft microdomains, rich in GM1 gangliosides and sphingomyelin, serve as specialised entrance sites for EVs. They aggregate signalling molecules such as Src family kinases and Gαq proteins, preparing target cells for rapid downstream activation upon vesicle fusion [147]. In hyperandrogenic PCOS, alterations in membrane lipid composition, including elevated ceramide-to-cholesterol ratios, modify bilayer curvature, facilitating the uptake of bigger extracellular vesicles (>200 nm) laden with inflammatory mediators through macropinocytosis.

Upon entering the receiving cell, EV trafficking employs various routing mechanisms to determine whether the cargo will be degraded, repurposed, or delivered intact to certain organelles [148]. Rab35 has emerged as a crucial regulator of extracellular vesicle docking to the plasma membrane recycling mechanism, ensuring the repeated presentation of particular receptors. This may elucidate the enduring vesicular signalling observed in granulosa cells of PCOS [149]. The retromer complex (VPS35–VPS26–VPS29) facilitates the movement of specific endosomal vesicles away from lysosomal degradation and towards retrograde transport to the trans-Golgi network. Post-translation, miRNAs and proteins can undergo modifications prior to exerting their effects [150]. In specific cases, EV cargo bypasses endosomal confinement by membrane destabilisation caused by fusogenic lipids (e.g., phosphatidylserine, phosphatidylethanolamine), subsequently releasing exosomal miRNAs straight into the cytosol to interact with Argonaute-loaded RISC complexes. lncRNAs carried by EVs can interact with heterogeneous nuclear ribonucleoproteins (hnRNPs), altering pre-mRNA splicing patterns in steroidogenic enzyme transcripts and consequently affecting the efficiency of androgen-to-estrogen conversion in ovarian tissue [151]. Protein cargo, including kinases, scaffolding proteins, and ubiquitin ligases, often localises to mitochondria, impacting fission and fusion dynamics via DRP1 and MFN2, which in turn affects ATP generation and the balance of ROS [152].

The signalling implications of electric vehicle adoption exceed conventional NF-κB and MAPK pathways, encompassing noncanonical routes and organelle-specific stress responses that connect metabolic, reproductive, and immunological dysfunctions. In granulosa cells, extracellular vesicle-associated Wnt ligands (Wnt5a, Wnt7b) activate both β-catenin-dependent and -independent pathways, influencing follicular cell proliferation, luteinisation potential, and steroidogenesis. In adipose tissue, miR-93-loaded extracellular vesicles suppress CDKN1A (p21), steering preadipocyte development towards hypertrophic, insulin-resistant adipocytes [153]. The hepatic absorption of EVs carrying sphingosine-1-phosphate (S1P) activates the S1PR2–RhoA–ROCK pathway, leading to enhanced gluconeogenesis and hepatic insulin resistance [154]. Skeletal muscle fibres that absorb EVs enriched in miR-486 have reduced PTEN levels, resulting in aberrant AKT activation and complications with AMPK-mediated mitochondrial biogenesis. This renders the body’s metabolism increasingly inflexible [155]. VEGFA-containing extracellular vesicles hyperactivate VEGFR2–PI3K–AKT–eNOS signalling in endothelial cells, first accelerating angiogenesis but subsequently destabilising blood vessels due to the presence of pro-apoptotic miR-34a [156].

Following EV cargo delivery, organelle–organelle contact introduces a significant degree of complexity. Mitochondrial dysfunction induced by EV-derived miR-210 or the inhibition of PGC-1α alters mitochondrial-associated ER membrane (MAM) signalling, impairing Ca^2+^ flux through IP3R–VDAC1 channels, which in turn affects steroid hormone biosynthesis in granulosa cells [157]. Excessive lipid-rich extracellular vesicles in lysosomes activate the TFEB/TFE3 transcription factors, accelerating lysosome biogenesis while altering autophagic flux, perhaps leading to the premature degradation of steroidogenic enzymes. The stimulation of p53–p21 stress responses in ovarian stroma by extracellular vesicles induces cellular senescence, hence augmenting inflammation through the release of senescence-associated secretory phenotypic factors. The senescence-inflammation loop alters follicular microvascularization and perpetuates insulin resistance and hyperandrogenemia via systemic circulation [158].

In obese PCOS, the absorption of extracellular vesicles and intracellular signalling mechanisms synergistically exacerbate reproductive and metabolic issues in many ways. EVs employ several mechanisms for cellular entry, intracellular transport, and signal transmission to organelles, ensuring the stable and accurate delivery of pathogenic instructions—such as regulatory RNAs, bioactive lipids, or enzymatic proteins—to sustain disease phenotypes [159]. The EV system is an excellent candidate for therapeutic intervention. Potential strategies encompass obstructing specific integrin-ECM interactions to inhibit targeted uptake, altering membrane lipid composition to modify preferences for EV internalisation, or selectively inhibiting Rab35- or retromer-mediated trafficking to cease prolonged vesicle-driven signalling while preserving essential EV communication for tissue homeostasis.

### 3.5. Systemic Crosstalk: From the Ovary to Peripheral Targets

The systemic communication between the ovary and peripheral organs in obese PCOS forms a complex, multi-layered signalling network in which EVs act as molecular couriers, delivering transcriptional regulators, metabolic modulators, and inflammatory initiators. This is a reciprocal process; ovarian dysfunction induces metabolic issues, while peripheral metabolic and inflammatory stress exacerbates ovarian complications [160]. Ovarian granulosa and theca cells, exposed to chronic hyperandrogenism, low-grade inflammation, and hyperinsulinemia, release EVs distinguished by a specific cargo profile, comprising small RNAs (e.g., miR-103, miR-125b, miR-21), lncRNAs, circRNAs, oxidised phospholipids, and DAMPs such as HMGB1 [161,162]. These vesicles infiltrate the bloodstream by exocytosis, a process that entails the ESCRT complex (TSG101, Alix) and RAB GTPases (RAB27A/B). This facilitates direct delivery to peripheral organs via interactions between integrins and ligands, as well as endocytosis mediated by lipid rafts [163].

Ovarian extracellular vesicles predominantly adhere to visceral adipocytes upon entering adipose tissue. Within these cells, miR-103 and miR-223 inhibit the functionality of IRS-1 and PI3K subunits while simultaneously augmenting SOCS3 expression [164]. This sequence of events inhibits PI3K–AKT–mTOR signalling, hence impeding GLUT4 vesicular trafficking and complicating insulin’s ability to uptake glucose [165]. Simultaneously, the transcriptional suppression of PPARγ and C/EBPα transforms adipocytes into a pro-lipolytic phenotype, resulting in the release of more FFAs into the bloodstream. Free fatty acids exacerbate systemic inflammation by activating TLR4, which initiates the MyD88–IRAK–TRAF6–NF-κB signalling cascade in hepatocytes and myocytes. This mechanism results in the phosphorylation of IRS proteins on serine residues, indicating insulin resistance at the molecular level.

In skeletal muscle, extracellular vesicles containing miR-29a, miR-34a, and protein phosphatase subunits (PP2A catalytic subunit) disrupt AMPK–PGC1α activation, hence diminishing mitochondrial biogenesis and oxidative phosphorylation capacity [166]. This induces a metabolic alteration that increases the prevalence of anaerobic glycolysis, resulting in elevated lactate production. Increased lactate/pyruvate ratios in systemic circulation alter cumulus–oocyte complex (COC) metabolism through monocarboxylate transporters (MCT1/MCT4) and modulate pyruvate dehydrogenase (PDH) activity, impeding mitochondrial ATP generation in oocytes [167]. Furthermore, when AMPK signalling is dysfunctional, it inhibits ULK1 activity, hence impeding autophagic flux. This complicates the elimination of malfunctioning mitochondria, resulting in an increased accumulation of ROS in muscle tissue, hence exacerbating systemic oxidative stress that impacts ovarian cells [168].

The liver is a significant target. The uptake of ovarian EV cargo, such as miR-122, miR-192, and protein tyrosine phosphatase 1B (PTP1B), activates endoplasmic reticulum stress pathways, particularly the PERK–eIF2α–ATF4–CHOP axis [169]. This enhances the production of PEPCK and G6Pase, hence facilitating gluconeogenesis, while concurrently diminishing the efficacy of insulin signalling. This alteration in hepatic food processing elevates blood glucose and VLDL particle levels, hence modifying the body’s lipid profiles [170]. Cholesterol-laden VLDL fractions provide excess steroidogenic substrates to theca cells and activate LXR–SREBP1c signalling in granulosa cells, worsening lipotoxicity and promoting mitochondrial membrane depolarisation in growing follicles [171].

Endothelial cells, functioning as systemic sentinels, internalise ovarian EVs loaded with miR-155, miR-21, ICAM-1, and VCAM-1 mRNA. These cargos activate NF-κB–p65, increase the adhesive properties of endothelial cells, and diminish the availability of NO by reducing eNOS levels and ET-1 concentrations [172]. Impaired nitric oxide-mediated vasodilation diminishes ovarian stromal perfusion, resulting in localised hypoxia and stabilisation of HIF1α, which modifies the expression of angiogenic factors (increased VEGF, decreased angiopoietin-1, increased angiopoietin-2), culminating in abnormal follicular vascularization and nutrient supply [173].

Monocytes and macrophages in the immune system internalise ovarian extracellular vesicles containing S100A8/A9 and oxidised cardiolipin. This initiates the NLRP3 inflammasome via a mitochondrial ROS–TXNIP interaction [174]. This results in robust secretion of IL-1β and IL-18, which directly inhibits FSH from inducing aromatase production in granulosa cells via the MAPK p38 and JNK pathways. This further enhances the steroidogenic equilibrium in favour of androgen production [175]. The same cytokines induce insulin resistance systemically by inhibiting insulin receptor signalling in the liver and skeletal muscle via SOCS3. This establishes a feedback loop that exacerbates the condition.

In reaction to these ovarian signals, peripheral organs produce their own EVs. Visceral adipose tissue secretes extracellular vesicles that encompass ceramides, miR-27a, and pro-inflammatory lipids. These EVs diminish the amounts of mitofusin-1/2 (MFN1/2) and ocular atrophy protein 1 (OPA1), disrupting the equilibrium between mitochondrial fusion and fission in oocytes. Hepatocyte-derived extracellular vesicles enriched in miR-122 and apolipoprotein fragments promote lipid accumulation in adipose tissues through SREBP1c–FASN activation, while muscle-derived extracellular vesicles containing myomiRs (miR-1, miR-206) regulate ovarian myofibroblast contractility, thereby indirectly affecting follicular rupture dynamics [176,177].

From a systems perspective, this EV-mediated communication between the ovary and periphery operates as a distributed endocrine–paracrine disease network, with vesicles acting as multi-cargo “molecular packages” that can synchronise transcriptional reprogramming, metabolic flux redirection, and inflammatory tone across multiple organs. This network is self-sustaining as each node—ovary, adipose tissue, liver, muscle, vasculature, immune system—modifies the extracellular vesicle cargo it disseminates to mirror both local stress conditions and systemic influences, hence perpetuating the disease. Disruption of any singular pathway—such as inhibiting ceramide synthesis in adipocytes, obstructing integrin-mediated extracellular vesicle uptake in ovarian stromal cells, or modulating RAB27A/B-mediated vesicle release—could theoretically recalibrate the entire network towards homeostasis, highlighting the therapeutic potential of targeting extracellular vesicle biology in obese polycystic ovary syndrome.

Table 1 delineates the bidirectional communication between the ovary and peripheral metabolic organs in obese PCOS mediated by EVs. It highlights the origin of EVs, their molecular components (microRNAs, proteins, lipids), target tissues, the primary intracellular signalling pathways involved, and the ensuing pathophysiological effects. Abbreviations: EV, extracellular vesicle; PCOS, polycystic ovary syndrome; PI3K, phosphoinositide 3-kinase; AKT, protein kinase B; mTOR, mechanistic target of rapamycin; PPARγ, peroxisome proliferator-activated receptor gamma; NF-κB, nuclear factor kappa-light-chain-enhancer of activated B cells; MFN, mitofusin; OPA1, optic atrophy protein 1; AMPK, AMP-activated protein kinase; PGC1α, peroxisome proliferator-activated receptor gamma coactivator 1-alpha; ULK1, unc-51 like autophagy activating kinase 1; ROS, reactive oxygen species; PERK, protein kinase RNA-like endoplasmic reticulum kinase; eIF2α, eukaryotic initiation factor 2-alpha; ATF4, activating transcription factor 4; LXR, liver X receptor; SREBP1c, sterol regulatory element-binding protein 1c; eNOS, endothelial nitric oxide synthase; NLRP3, NOD-, LRR-, and pyrin domain-containing protein 3; MAPK, mitogen-activated protein kinase.

Table 1 summarizes the pathways of systemic crosstalk in PCOS. The molecular contents of EVs and their subsequent impacts on adipose tissue, skeletal muscle, liver, endothelium, and the immune system are shown in the table, which also examines the role of EVs in facilitating communication between the ovary and peripheral organs.

Table 1 shows the interplay between the ovary and peripheral metabolic organs in obese PCOS is increasingly recognised as being modulated by EVs that encompass diverse molecular components. Extracellular vesicles from granulosa and theca cells transport miRNAs such as miR-103 and miR-21, along with compounds associated with oxidative stress, to adipose, hepatic, muscular, and endothelial destinations [178]. This alters pathways such as PI3K–AKT–mTOR, NF-κB, and PPARγ signaling, resulting in insulin resistance, systemic inflammation, and modifications in lipid metabolism [179]. Conversely, extracellular vesicles from adipose tissue enriched with miR-27a and ceramides target ovarian steroidogenic cells, diminishing aromatase synthesis and exacerbating hyperandrogenism. EVs produced from skeletal muscle influence oocyte mitochondrial activity by inhibiting the AMPK–PGC1α and ULK1 signaling pathways [180]. Hepatic extracellular vesicles influence ovarian metabolism by inducing endoplasmic reticulum stress (PERK–eIF2α–ATF4) and promoting lipogenesis (LXR–SREBP1c) [181]. Endothelial EVs impair ovarian angiogenesis by downregulating eNOS and upregulating endothelin-1, whereas immune cell-derived EVs activate the NLRP3 inflammasome in granulosa cells, so linking systemic inflammation to ovarian dysfunction. This EV-mediated molecular interaction links metabolic dysregulation to ovarian complications in obese PCOS, paving the door for the development of diagnostics and targeted therapies [182].

Understanding the molecular constituents and signalling pathways that enable EV-mediated communication between the ovary and peripheral metabolic organs presents new avenues for therapeutic intervention in obese PCOS. Focusing on adipose-derived EVs enriched with miR-27a and ceramides may reinstate ovarian aromatase activity and alleviate androgen surplus, potentially through miRNA antagonists or regulators of ceramide formation. Modifying the hepatic EV cargo via small-molecule inhibitors that target the PERK–eIF2α–ATF4 pathway or medicines that reduce SREBP1c activation may alleviate ovarian lipotoxicity and improve folliculogenesis. Pharmacological AMPK activators or PGC1α enhancers may mitigate the impact of skeletal muscle-derived EVs on oocyte mitochondrial integrity by reinstating oxidative phosphorylation and averting mitochondrial fragmentation. In endothelial-ovarian communication, restoring eNOS expression with L-arginine supplementation, statin medication, or phosphodiesterase inhibitors may normalize ovarian angiogenesis. The activation of the NLRP3 inflammasome in granulosa cells driven by immune-derived extracellular vesicles presents a promising target, with new NLRP3 inhibitors or IL-1β antagonists potentially alleviating localized inflammation. Furthermore, therapeutic strategies utilising modified EVs that incorporate beneficial microRNAs (e.g., miR-126 for angiogenesis, miR-320 for metabolic control) or anti-inflammatory compounds may be developed as precision medicines for PCOS. Integrating these strategies into weight reduction programs, lifestyle modifications, and insulin-sensitizing drugs may yield a synergistic effect, directly targeting both metabolic and reproductive issues in obese women with PCOS.

### 3.6. Therapeutic and Diagnostic Potential of EV in Obese PCOS

The therapeutic and diagnostic application of EV in obese PCOS relies on their unique biogenesis and bioactive composition, which encapsulate the molecular markers of metabolic and reproductive dysfunction [183]. In the diagnostic domain, EVs encompass an array of miRNAs, lncRNAs, circRNAs, proteins, lipids, and metabolites that elucidate cellular and tissue alterations in obese PCOS. Dysregulated miRNAs, including as miR-122, miR-27b, miR-21, miR-103, miR-222, and miR-320, are frequently found to be either elevated or diminished in circulating EVs [133]. This influences lipid metabolism (via the regulation of SREBP1 and FASN), insulin signalling (by influencing PI3K–Akt–mTOR and AMPK), and steroidogenic activity (by targeting CYP17A1 and HSD3B2) [184]. Quantitative EV miRNA profiling by droplet digital PCR, next-generation sequencing, or NanoString technology provides enhanced sensitivity for the early identification of subclinical metabolic–reproductive imbalances, surpassing traditional static hormonal testing [185]. Proteomic extracellular vesicle signatures, featuring the enrichment of adiponectin receptor fragments, phosphorylated AKT, and SOCS3, enhance diagnostic specificity by directly linking systemic insulin resistance to intra-ovarian signalling abnormalities [186].

In therapy, EVs have two potential functions: (1) as naturally occurring bioactive agents that facilitate intercellular communication, and (2) as manufactured nanocarriers for the precise delivery of molecular therapies [187]. In PCOS, adipose tissue-derived extracellular vesicles often exacerbate ovarian inflammation by transmitting pro-inflammatory microRNAs (e.g., miR-155, which targets SHIP1 and promotes NF-κB activation); hence, counteracting this signal using modified extracellular vesicles is a viable method [188]. When loaded with antagomirs targeting miR-122, extracellular vesicles from hepatocytes can inhibit lipogenesis driven by SREBP1. This diminishes VLDL secretion from the liver and indirectly alleviates ovarian hyperandrogenism by reducing systemic insulin levels [189]. Conversely, extracellular vesicles from granulosa cells abundant in let-7 family miRNAs can enhance the sensitivity of theca cells to insulin by inhibiting IRS2–PI3K hyperactivation, which would otherwise lead to excessive androgen production. The administration of miR-320 mimics via extracellular vesicles may suppress MAPK–ERK–c-Fos signalling, promoting follicular cell growth and reducing apoptosis [190].

Advanced EV surface engineering approaches, such as the modification of EV membranes with anti-FSHR antibodies, LHR-targeted peptides, or hyaluronic acid ligands, promote selective targeting of ovarian granulosa and theca cells, thereby markedly improving on-target delivery [191,192]. Upon internalisation by the cell, EVs discharge their contents into the cytoplasm via endosomal escape or membrane fusion, precisely altering intracellular signalling pathways. This targeted method is especially advantageous in obese PCOS, where systemic medication administration may result in off-target effects due to widespread insulin resistance and chronic inflammation.

In addition to altering miRNAs, EV-mediated protein delivery possesses therapeutic potential. EVs from MSCs containing functional SIRT1 protein can activate the AMPK–PGC1α–NRF1 pathway in granulosa cells, promoting mitochondrial biogenesis, enhancing oxidative phosphorylation, and reducing the accumulation of reactive oxygen species [193]. Similarly, EVs incorporating catalase or SOD2 proteins might directly alleviate the increased oxidative stress seen in the PCOS ovarian milieu, therefore improving meiotic spindle integrity and oocyte competence [194]. Preclinical findings indicate that extracellular vesicles containing VEGF-A and angiopoietin-1 promote angiogenesis in the ovarian stroma by activating the PI3K–Akt–eNOS pathway, hence restoring vascularization and nutrition supply to follicles [195]. From a regenerative perspective, MSC-EVs exhibit anti-apoptotic and pro-survival effects by modifying Bcl-2/Bax ratios through the JAK2–STAT3 and ERK1/2 pathways. They inhibit the apoptosis of granulosa cells by preventing the formation of the NLRP3 inflammasome, hence preserving follicular viability. The anti-fibrotic capacity of EVs is achieved by the suppression of TGF-β1–SMAD2/3 signalling, therefore preventing excessive extracellular matrix deposition in the ovarian cortex, a phenomenon observed in advanced PCOS disease [196].

The integration of EV biomarkers into diagnostic algorithms in clinical practice may enhance the molecular phenotyping of obese patients with PCOS. Machine learning methodologies utilised on EV-derived multi-omic datasets—encompassing transcriptomic, proteomic, and lipidomic profiles—may classify patients into metabolic–inflammatory, androgen-dominant, or mixed phenotypes, predicting differential responses to pharmacological (e.g., metformin, GLP-1 receptor agonists) or lifestyle interventions. This form of phenotyping may identify individuals most likely to benefit from EV-based therapy, hence accelerating the advancement towards precision reproductive endocrinology. To implement EV-based therapeutics in clinical practice, it is essential to enhance isolation (size-exclusion chromatography, tangential flow filtration, immunoaffinity capture), characterisation (nanoparticle tracking analysis, flow cytometry, transmission electron microscopy), and quality control protocols to comply with Good Manufacturing Practice standards. Discussing scalability, reproducibility, and biosafety is crucial, particularly for immunogenicity, long-term biodistribution, and the potential for horizontal gene transfer of EV payload. Upon overcoming these regulatory and technical obstacles, EVs may evolve from promising experimental tools to standard molecular interventions for obesity-related PCOS, establishing a unique link between the syndrome’s molecular pathology and customised, targeted therapeutic strategies. Table 2 highlights the diagnostics and therapeutics strategies.

Recent breakthroughs in EV biology have accelerated its transformation into diagnostic and therapeutic applications for obese PCOS. Circulating extracellular vesicle microRNA panels (e.g., miR-122, miR-27b, miR-21, miR-103, miR-222, miR-451) and protein signatures (e.g., phosphorylated AKT, SOCS3, adiponectin receptor fragments) can enhance the early identification of metabolic–reproductive dysregulation and the classification of insulin resistance-dominant phenotypes. The cargo of EV in follicular fluid, comprising miR-21, miR-93, and lncRNA H19, holds prognostic value for oocyte competence and outcomes in ART, especially when integrated with evaluations of FSHR–cAMP–PKA signalling and mitochondrial function [119]. Urinary EVs carrying persistent renal microRNAs (e.g., miR-21, miR-125b) may function as a non-invasive biomarker for HA and inflammation in obese patients with PCOS. From a medicinal standpoint, engineered hepatocyte-derived EVs harbouring anti-miR-122 antagomirs have the ability to modulate hepatic lipid metabolism (via SREBP1c suppression), reduce systemic insulin levels, and indirectly alleviate ovarian hyperandrogenism. However, the application in clinical practice requires standardised extracellular vesicle extraction, optimisation of stability, and comprehensive evaluation of biodistribution, off-target effects, and reproducibility among cohorts.

## 4. Clinical Implications and Translational Perspectives

### 4.1. Integrating EV Biomarkers into PCOS Risk Stratification

The integration of EV-derived biomarkers into clinical protocols for PCOS represents a significant progression in personalized medicine, especially for PCOS accompanied with obesity, particularly in phenotypes characterised by hyperandrogenism and metabolic dysfunction, marked by the presence and intensification of complex metabolic and reproductive disorders [197]. Traditional PCOS classification systems, such as the Rotterdam and NIH criteria, primarily rely on clinical manifestations—hyperandrogenism, oligo/anovulation, and polycystic ovarian morphology—combined with biochemical indicators including serum androgen levels, fasting insulin concentrations, HOMA-IR values, and dyslipidaemia [198]. While these indications possess therapeutic relevance, they insufficiently reflect the molecular heterogeneity linked to PCOS, especially the intricate inter-organ connection involving adipose tissue, the ovary, liver, skeletal muscle, and immune systems. Molecular cargo from EVs, such as miRNAs, lncRNAs, mRNAs, lipids, and proteins, can serve as minimally invasive molecular indicators that reflect the current state of systemic metabolic pathways and the ovarian microenvironment. Incorporating these into clinical decision-making may produce stratification models that are both mechanistically robust and temporally adaptable, accurately reflecting disease dynamics in ways that static biochemical testing cannot accomplish.

From a biological standpoint, unique extracellular vesicle miRNA expression patterns are increasingly predictive of polycystic ovary syndrome phenotypic differentiation and associated metabolic risk profiles. An elevation in EV-associated miR-122, miR-34a, and miR-27b in obese PCOS has been correlated with enhanced hepatic de novo lipogenesis via the activation of sterol regulatory SREBP1c, resulting in triglyceride accumulation and insulin resistance in the liver [199]. Simultaneously, elevated concentrations of miR-103 and miR-107 in extracellular vesicles impede the phosphorylation of IRS-1 on tyrosine, hence inhibiting the activation of PI3K–AKT in skeletal muscle [200]. This diminishes the efficacy of GLUT4 translocation and exacerbates systemic insulin resistance. Conversely, EVs originating from FF that are abundant in miR-21, miR-93, miR-132, and miR-222 exert direct influences on granulosa cell proliferation, aromatase (CYP19A1) activity, and mitochondrial membrane potential via pathways governed by transforming growth factor-beta (TGF-β)/SMAD, AMPK–mechanistic target of rapamycin (mTOR), and peroxisome proliferator-activated receptor gamma coactivator 1-alpha (PGC-1α) [201,202]. The FF-EV molecular fingerprints can be computationally weighted into composite indices that differentiate between insulin resistance-dominant, hyperandrogenism-dominant, or mixed PCOS subtypes. This is crucial for optimising the efficacy of focused therapeutic approaches [203].

Utilising EV biomarker assays in PCOS workflows necessitates a multi-step procedure. This entails employing standardised extracellular vesicle isolation techniques such as ultracentrifugation, size-exclusion chromatography, or immunoaffinity capture, in conjunction with high-throughput molecular quantification platforms including NGS, digital droplet PCR (ddPCR), or targeted mass spectrometry for protein cargo analysis [204]. Quantitative normalisation by NTA or tunable resistive pulse sensing (TRPS) ensures that changes in extracellular vesicle molecular profiles are attributable to disease processes rather than fluctuations in vesicle concentration [205]. Advanced bioinformatics and machine learning models can integrate extracellular vesicle cargo data with anthropometric indices (BMI, WHR), metabolic markers (fasting insulin, HOMA-IR, triglycerides), and reproductive endpoints (antral follicle count, AMH levels, oocyte retrieval outcomes) to generate personalised, multi-dimensional risk scores [197]. An obese PCOS patient displaying increased circulating EV miR-122 and miR-34a, alongside reduced EV miR-451 and heightened BMI/WHR ratios, may be classified within a high-risk cluster for hepatic steatosis with associated insulin resistance-induced reproductive dysfunction, warranting early combination therapy that includes metabolic modulation (e.g., GLP-1 agonists) and ovulatory support [206].

The prolonged application of EV biomarker monitoring introduces a dynamic dimension to PCOS risk classification, enabling physicians to observe alterations in molecular levels in response to lifestyle modifications, pharmacological treatments, or assisted reproductive techniques [207]. Reductions in circulating extracellular vesicle miR-122 and miR-27b levels following structured weight-loss interventions may be associated with the reactivation of AMPK phosphorylation, the inhibition of ACC activity, and the downregulation of hepatic lipogenesis, suggesting an enhancement in metabolic health. Similarly, the normalisation of FF-EV miR-21 and miR-93 after metformin treatment may indicate the reinstatement of granulosa cell mitochondrial function, a decrease in oxidative stress, and improved folliculogenesis [106,208]. Conversely, persistently elevated levels of pro-inflammatory extracellular vesicle cargo, such as exosomal proteins associated with TNF-α or miRNAs modulated by NF-κB (including miR-155 and miR-146a), may indicate the presence of chronic low-grade inflammation and oxidative stress [209]. This indicates that more antioxidant interventions or specific anti-inflammatory medications may be necessary.

Ultimately, integrating EV-derived biomarker panels into diagnostic and risk stratification algorithms for PCOS could facilitate a shift from symptom-based classification to dynamic, systems biology-focused precision therapy. This paradigm would facilitate the prediction of disease progression to type 2 diabetes mellitus, non-alcoholic fatty liver disease, or cardiovascular issues, while also enhancing the likelihood of a successful pregnancy. By leveraging the molecular specificity and real-time responsiveness of extracellular vesicle cargo, doctors could tailor therapies to correspond with the patient’s current phenotype and projected trajectory, thereby improving both metabolic and reproductive outcomes in obese PCOS populations.

### 4.2. EV-Based Interventions in ART

Employing EV-based approaches in ART for obese PCOS patients offers an innovative solution to address the intrinsic deficits in oocyte and embryo quality that often impair fertilisation efficiency and implantation potential. In obese PCOS, the follicular milieu is characterised by chronic low-grade inflammation, increased ROS, altered steroidogenic signalling, and metabolic stiffness, all of which impair the granulosa–cumulus–oocyte communication network [210]. In this domain, EVs serve as significant molecular communicators. In the obese PCOS phenotype, the cargo frequently comprises pro-inflammatory miRNAs (such as miR-155 and miR-146a), anti-mitochondrial lncRNAs, and proteins that disrupt INSR and gonadotropin receptor signalling pathways. This pathogenic EV content maintains NF-κB activation abnormally, reduces AMPK phosphorylation, and disrupts the PI3K–AKT–mTOR–FOXO1 regulatory pathways. This complicates the oocyte’s acquisition of metabolic substrates and its maturation within the nucleus [199]. Therapeutic modulation of EV signaling—through the removal of detrimental EV populations, the enhancement with beneficial EVs, or the engineering of vesicles with targeted molecular payloads—could re-establish homeostasis in the ovarian follicle, thereby increasing the production of developmentally competent MII oocytes and improving subsequent embryo viability [211].

At the molecular intervention level, tailored EVs can be altered to deliver cargo that directly reinstates the damaged signalling pathways in follicles impacted by obesity-related PCOS. MSC-EVs abundant in miR-320 and miR-21 mimics can inhibit apoptosis by decreasing pro-apoptotic BAX and caspase-3 levels while increasing anti-apoptotic BCL-2 levels. This occurs via modifications to the MAPK–ERK–c-Fos pathway [212]. The concurrent delivery of miR-132 and miR-212 through EVs can augment the expression of StAR and normalise CYP19A1 aromatase activity, thereby reinstating the estradiol/testosterone (E2/T) ratios critical for synchronised granulosa cell proliferation and the integrity of the COC. Alternative methods for engineering EV cargo involve including antioxidant enzymes such as catalase, GPx, or SOD2 into the EVs [213]. These enzymes mitigate mitochondrial reactive oxygen species, stabilise the mitochondrial membrane potential (ΔΨm), and prevent spindle abnormalities induced by oxidative microtubule damage. The PGC-1α–TFAM axis can be triggered via the EV-mediated transfer of certain lncRNAs or fragments of transcription factors [214]. This can additionally promote mitochondrial biogenesis, which is crucial for ATP production necessary for the energy-demanding activities of meiotic progression, cytoskeletal remodelling, and spindle orientation. In preclinical settings, these mitochondrial rescue effects have been directly associated with enhanced MII oocyte maturation rates and better blastocyst formation efficiency.

In the context of ART laboratories, the enhancement of IVM and fertilisation media with EVs sourced from healthy donor follicular fluid or optimised MSC cultures has been shown to improve the functional efficacy of COCs [215]. EV supplementation activates the PI3K–AKT–mTOR and AMPK–SIRT1 signalling pathways concurrently, resulting in increased glucose uptake mediated by GLUT1/GLUT3, enhanced fatty acid oxidation through CPT1A, and improved mitochondrial oxidative phosphorylation (OXPHOS) efficiency [216]. The cumulative consequence is a metabolic alteration that increases energy availability, reduces meiotic arrests, and facilitates correct chromatin condensation. Moreover, EVs rich in angiogenic factors such as VEGF-A and IGF-1 promote the remodelling of ovarian microvessels via PI3K–AKT–eNOS–NO signalling, enhancing blood flow to the follicles, nutrient delivery, and waste product disposal. Simultaneously, GDF9 and BMP15 conveyed via EVs can re-establish bidirectional signalling between oocytes and granulosa cells, ensuring concurrent cytoplasmic and nuclear maturation [217].

Interventions utilising extracellular vesicles extend beyond oocyte maturation and influence embryonic development post-fertilization. Embryos from obese persons with PCOS often exhibit altered morphokinetics, marked by delayed cleavage, uneven blastomere divisions, and increased fragmentation rates. Incorporating EVs that include components of the miR-17-92 cluster, which regulate cyclin D1/CDK4 activity, can rectify the cell cycle, safeguard the genome, and diminish the likelihood of aneuploidy by enhancing SAC regulation [218]. Similarly, EVs enriched with anti-apoptotic lncRNAs such as H19 and MALAT1 can inhibit mitochondrial-mediated apoptotic pathways by preventing the release of cytochrome c and the activation of caspases. This preserves the integrity of the blastocyst structure and increases the quantity of high-quality trophectoderm cells. At the epigenetic level, EV-mediated transfer of miRNAs regulating DNMT3A and DNMT3B can rectify aberrant DNA methylation patterns in embryos derived from PCOS, potentially enhancing implantation success and reducing the risk of long-term developmental programming abnormalities [219].

A meticulous strategy is required to transform EV-based ART interventions into practical therapies. Prior to ovarian stimulation, individualised profiling of circulating and follicular fluid-derived extracellular vesicle cargo can identify deficiencies—such as an absence of mitochondrial-supportive microRNAs, an elevation in pro-inflammatory cytokine-associated extracellular vesicle proteins, or alterations in lipid metabolism regulators—that facilitate the development of tailored extracellular vesicle formulations [135]. Advanced bioengineering techniques enable the modification of therapeutic EVs to display ligands for either the FSHR or the LHR. This ensures that granulosa and theca cells absorb them while minimising systemic exposure to inappropriate cells. Furthermore, live imaging methodologies such as intravital microscopy and nanoparticle-based tracking can be employed to observe the movement of extracellular vesicles within the ovaries during assisted reproductive technology cycles. This provides real-time evidence of tissue-specific delivery. Regulatory approval and reproducibility among patient populations will rely on such customization tactics, in conjunction with robust GMP-compliant manufacturing processes and standardized dosage protocols [220].

Integrating these molecularly precise EV-based techniques into ART workflows could possibly transform treatment paradigms for obese PCOS patients. These medicines directly alter the molecular environments of the ovaries and embryos, addressing the fundamental issue rather than merely modifying hormones throughout the body. This may lead to enhanced oocyte competence, increased fertilisation rates, improved embryo quality, superior implantation results, and a greater number of live births. The future clinical applicability of EV-based ART interventions will rely on closing the divide between preclinical efficacy and extensive human trials, determining long-term safety profiles, and incorporating these tools into personalised reproductive medicine frameworks that account for both metabolic and reproductive outcomes.

### 4.3. Personalized Therapeutics: Targeting Metabolic–Reproductive Axis Dysfunction

The transition to personalised treatment for obese PCOS relies on integrating high-resolution molecular diagnostics, particularly EV transcriptomic, proteomic, and lipidomic markers, with targeted therapies aimed at the disturbed metabolic–reproductive axis. In obese PCOS, this axis is disrupted by interconnected feedback loops: IR exacerbates HA by overstimulating theca cells, HA aggravates IR through interactions with adipocyte androgen receptors, and both contribute to chronic inflammation mediated by NF-κB, which inhibits glucocorticoid steroidogenesis. In this condition, extracellular vesicles serve as a mechanistic conduit, transporting pro-inflammatory cytokine transcripts, miRNAs associated with mitochondrial impairment, and metabolic regulators among the adipose, hepatic, and ovarian compartments. This cargo alters the PI3K–AKT–mTOR, AMPK–SIRT1–PGC-1α, and TGF-β/SMAD pathways, affecting the localisation of FOXO1 inside the nucleus, the phosphorylation of GSK3β, and the downstream gene expression critical for folliculogenesis. Personalised treatment solutions must involve both systemic control of glucose and lipid metabolism and localised correction of follicular and endometrial molecular settings, aligning metabolic restoration with reproductive time.

Molecular phenotyping utilising extracellular vesicles facilitates patient stratification that surpasses clinical indicators like BMI, WHR, or LH/FSH ratio. For example, elevating the concentrations of EV miR-27b, miR-122, and miR-34a, which are recognised for activating ChREBP and SREBP1c, aids in identifying patients whose metabolic disorders are mostly attributable to hepatic lipogenesis rather than AMPK activation. In certain cases, GLP-1 receptor agonists or biguanides can restore AMPK-mediated ACC phosphorylation, hence reducing malonyl-CoA buildup and enhancing CPT1A-dependent β-oxidation. Conversely, EV profiles defined by miR-155, miR-21, and IL-6 mRNA indicate systemic NF-κB activation, leading to p65–p50 nuclear translocation and the overexpression of the pro-apoptotic protein BAX in GCs. Such patients may derive advantages from the early utilisation of SPMs that activate ALX/FPR2 or ChemR23 receptors. This halts the inflammatory cascade by reducing IKKβ phosphorylation. A distinct subgroup, marked by increased levels of miR-93 and miR-132 that suppress CYP19A1 expression, may require specific aromatase regulation to normalise E2/T ratios, thus restoring ERα/ERβ signalling and COC expansion dynamics. Significantly, these interventions may be integrated with antioxidant methods, such as peptides that specifically target mitochondria, to maintain ΔΨm stability and spindle integrity throughout the transition from MII to MII.

In ART cycles, pre-stimulation EV analysis can facilitate protocol selection and the accurate dosage of gonadotropins at the molecular level. Increased concentrations of EV miR-200a/b/c, which enhance ZEB1/2 suppression and raise LHR expression, may predispose individuals to early luteinisation, highlighting the need for antagonist procedures with modified trigger timing. Reduced levels of EV miR-451 and miR-320, crucial for GLUT1/GLUT3-mediated glucose uptake and PGC-1α–TFAM-driven mitochondrial replication, indicate that metabolic priming with CoQ10, nicotinamide riboside, or targeted MSC-EVs enriched with mitochondrial enhancers is necessary prior to stimulation initiation. This synchronisation ensures that critical meiotic milestones, such as GVBD, spindle assembly, and the transition from metaphase to anaphase, occur under optimal bioenergetic and redox circumstances. Furthermore, angiogenic deficiencies resulting from EVs (e.g., reduced VEGF-A, Ang1) can be remedied through the EV-mediated delivery of pro-angiogenic factors, improving ovarian stromal perfusion via PI3K–AKT–eNOS–NO activation, thereby enhancing follicular oxygenation and nutrient supply during stimulation.

Personalised treatments can employ modified EVs as direct molecular delivery mechanisms. Autologous extracellular vesicles can be augmented ex vivo with the application of AMPK-activating microRNAs (miR-451a, miR-223), antioxidant enzymes (SOD2, GPx, CAT), and anti-apoptotic long non-coding RNAs (H19, MALAT1). Subsequently, they can be functionalised with ligands that specifically target FSHR or αvβ3-integrin, enabling selective uptake by GCs, CCs, or endometrial stromal cells. EV co-loading techniques can yield synergistic payloads, such as miR-17-92 to enhance cyclin D1/CDK4 activity in conjunction with CPT1A activators to augment β-oxidation, so ensuring simultaneous enhancement of mitochondrial energy supply and cell cycle integrity. Timing is paramount: follicle-targeting EVs should be administered during the early antral phase to prime the follicular niche for exogenous gonadotropin exposure, whereas endometrium-targeting EVs should be delivered during the peri-implantation window to modulate the Wnt/β-catenin and LIF–STAT3 pathways, enhancing endometrial receptivity and facilitating trophoblast adhesion.

These therapies require continual input from continuing EV and endocrine monitoring. Continuous EV profiling throughout medication can track early molecular adjustments—such as re-established AMPK phosphorylation, inhibition of p65 nuclear translocation, and normalisation of CYP19A1 mRNA—that occur prior to enhancements in ovulation rates, oocyte quality, or blastocyst morphology. Integrating endocrine (AMH, AFC) and metabolic (HOMA-IR, lipid panel) markers enables the development of real-time treatment response models that facilitate prompt protocol adjustments. This approach transforms therapy into an adaptable system capable of evolution throughout time. Each intervention is employed, assessed at the molecular level, and modified according to continuous EV biomarker feedback. This ensures the simultaneous optimisation of reproductive and metabolic objectives. The final output is a closed-loop precision medicine system where molecular biology informs ART decisions, resulting in improved pregnancy outcomes and reduced long-term metabolic risk.

## 5. Discussion

### 5.1. Synthesis of Molecular and Clinical Evidence

A substantial body of evidence indicates that EVs play a crucial role in the communication across metabolic, reproductive, and inflammatory processes in obese PCOS. They function as nanoscale carriers for the lateral transfer of regulatory RNAs, proteins, and lipids. Duval et al. provided a comprehensive synthesis of the altered EV cargo profile in PCOS, namely in FF, where specific miRNA clusters, particularly the miR-379 and miR-200 families, are persistently dysregulated. These miRNAs are recognised for their modulation of critical molecular pathways such as PI3K–AKT, MAPK–ERK, and TGF-β–SMAD, which regulate the proliferation of GC, the enlargement of cumulus cells, and the capacity of oocytes to undergo meiosis. Impairment of PI3K–AKT signalling might hinder glucose uptake and ATP production in the mitochondria, adversely affecting the metabolic support of the COC. Malfunctioning MAPK–ERK signalling can inhibit LH-induced meiotic resumption. Modified TGF-β–SMAD signalling can similarly impair ECM remodelling in the peri-ovulatory follicle, leading to postponed oocyte release and diminished zona pellucida integrity.

Furthermore, Vogt et al. discovered that women with PCOS exhibiting IR had significantly reduced levels of miR-320a in their bloodstream [20]. The reduction in miR-320a levels was associated with increased clinical pregnancy rates in ART cycles. Notably, the expression of miR-320a was shown to be affected by androgen levels, suggesting hormonal modulation of its production and incorporation into circulating EVs. At the molecular level, miR-320a modulates the phosphorylation of IRS, enhances the integrity of the PI3K–AKT signalling pathway, and alters the translocation of GLUT4 in peripheral tissues, hence influencing systemic insulin sensitivity [20]. In the reproductive axis, miR-320a-enriched extracellular vesicles may regulate FSH-responsive genes in granulosa cells, enhance steroidogenic enzyme expression, and influence anti-apoptotic pathways through BCL-2 family proteins [106]. In conclusion, these findings indicate a scenario wherein modified EV-derived miRNA profiles connect metabolic failure with ovarian signalling, resulting in the reproductive phenotype of obese PCOS through synchronised alterations in both systemic and follicular microenvironments.

An integrated review of molecular and clinical evidence reveals that EV-mediated communication in obese PCOS represents a multi-organ signalling network rather than a unique tissue occurrence [221]. Adipose tissue, skeletal muscle, liver, hypothalamus, and ovary exchange EVs that provide distinct signals on metabolism and reproduction. This network maintains reproductive homeostasis under metabolically healthy settings by regulating food availability, hormone signals, and follicular growth. In obese PCOS, the EV message is pathologically altered, incorporating increased pro-inflammatory cytokines, lipotoxic metabolites, and dysregulated miRNAs, which disrupt both systemic and local signalling [106,222].

At the systemic level, modified extracellular vesicle cargo results in hyperinsulinemia, exacerbating androgen excess via elevating CYP17A1 and diminishing aromatase in the ovary [223]. Inflammatory miRNAs (e.g., miR-21, miR-155) transported by adipose-derived extracellular vesicles activate NF-κB in granulosa and theca cells, perpetuating cytokine secretion and further disrupting PI3K–AKT and AMPK signalling pathways [224]. In skeletal muscle, impaired EV-mediated transport of insulin-sensitizing miRNAs (e.g., miR-320a, miR-99a) obstructs GLUT4 trafficking and reduces glycogen synthesis, hence worsening systemic insulin resistance. These metabolic imbalances influence the HPO axis, altering the pulsatility of GnRH and the ratios of LH to FSH, which subsequently impacts the recruitment and maturation of follicles [225].

This EV-mediated disease manifests clinically as reduced oocyte quality, lower fertilisation rates, and impaired endometrial receptivity [226]. Research demonstrates that follicular fluid-derived extracellular vesicles from patients with polycystic ovary syndrome display altered profiles of steroidogenic regulators, metabolic enzymes, and oxidative stress modulators, correlating with unsatisfactory assisted reproductive technology outcomes [227]. Diminished GPX4 and SOD1 cargo in FF-EVs correlates with increased ROS levels in the follicular niche, hence elevating the likelihood of mitochondrial dysfunction in oocytes. Furthermore, the existence of miRNAs that inhibit VEGF signalling may obstruct angiogenesis in the peri-ovulatory follicle, thus restricting the supply of nutrients and oxygen to the developing egg. These genetic anomalies underscore the prospective significance of EV profiles as diagnostic biomarkers and therapeutic targets [228].

### 5.2. Therapeutic Modulation of Extracellular Vesicles in Polycystic Ovary Syndrome

The therapeutic manipulation of extracellular vesicle signalling in obese polycystic ovary syndrome is rapidly gaining prominence as an effective approach to concurrently address metabolic, inflammatory, and reproductive issues [212]. Interventions can be delineated through three interrelated strategies: (1) suppressing the release of pathogenic EVs from metabolic tissues, (2) reconfiguring EV cargo profiles to reinstate physiological signalling, and (3) utilising engineered EVs as targeted delivery mechanisms to the ovary, hypothalamus, or liver [229]. The theoretical framework for these interventions is based on the understanding that EVs act as “molecular integrators,” conveying a mixture of microRNAs, proteins, and bioactive lipids that affect insulin sensitivity, steroidogenesis, mitochondrial function, and inflammatory responses in different organ systems [230]. In obese PCOS, the pathogenic extracellular vesicle phenotype is characterised by a prevalence of inflammatory microRNAs (e.g., miR-21, miR-155), lipotoxic ceramides, and oxidised phospholipids that activate NF-κB and JAK–STAT signalling in granulosa cells, hepatocytes, and adipocytes [190]. Inhibiting their release, via lifestyle changes, pharmacological interventions, or bariatric procedures, can reduce systemic low-grade inflammation and improve reproductive endocrinology by restoring the balance between PI3K–AKT activation and AMPK phosphorylation in metabolic and reproductive tissues [231].

Castaño et al. demonstrated that therapeutic alteration of EV-miRNA cargo, achieved through targeted manipulation of metabolic status, can significantly decrease hepatic lipogenesis and improve systemic insulin sensitivity [232]. The reprogramming of EV cargo resulted in the downregulation of lipogenic transcription factors SREBP1 and ChREBP, as well as critical enzymes such as FASN and ACC1, while simultaneously enhancing AMPK activation in hepatocytes. This simultaneous action reduced de novo lipogenesis and increased fatty acid oxidation, mitigating NAFLD and lowering circulating triglycerides, which in turn decreases hyperinsulinemia and ovarian androgen production [232]. Núñez López et al. revealed this metabolic axis by revealing that fluctuations in EV-miR-374b-5p levels were substantially correlated with increases in HbA1c, fasting glucose, and HOMA-IR [233]. Mechanistically, miR-374b-5p regulates components of the insulin receptor signalling cascade, including the phosphorylation state of IRS-1 and the consequent activation of AKT, thereby promoting the translocation of GLUT4 in skeletal muscle and adipose tissue. These findings confirm EV-miRNAs as biomarkers for metabolic improvement and as active molecular agents in reversing insulin resistance in obese PCOS [234].

Preclinical evidence strongly supports the use of exogenous EV supplementation in the reproductive and ovarian milieu to alleviate the pathological changes induced by PCOS. Fang et al. found that exosomes generated from brown adipose tissue (BAT-Exos), delivered intravenously to letrozole-induced PCOS mice, effectively normalised oestrous cyclicity, reduced LH and testosterone levels, and enhanced ovulation and conception rates [235]. BAT-Exos suppressed STAT3 phosphorylation, thereby diminishing IL-6 and TNF-α transcription in GCs, while simultaneously elevating GPX4 expression to prevent ferroptotic cell death. This antioxidant effect maintained mitochondrial membrane potential, increased ATP generation, and protected against excessive lipid peroxidation—factors directly linked to improved oocyte competence [235]. Jiang et al. provided more insights by demonstrating that plasma exosomes from PCOS patients alter the oestrous cycle and steroid hormone secretion in recipient mice, indicating that dysregulated EV cargo may interfere with the HPO axis [176]. These findings underscore the therapeutic potential of correcting EV profiles to restore local follicular signalling and systemic endocrine balance.

Cell-based EV treatments have emerged as a particularly promising translational avenue. Park et al. demonstrated that exosomes derived from MSCs, either via intravenous infusion or intraovarian injection, significantly improved glucose metabolism in PCOS animal models, restored ovarian morphology to normalcy, and reinitiated follicular development [223]. Mechanistically, MSC-EVs modulated the TGF-β–SMAD pathway to enhance GC proliferation, reduced apoptotic signalling by downregulating BAX and cleaved CASPASE-3, and increased the expression of steroidogenic enzymes (CYP19A1, HSD17B1) to promote oestrogen production [223]. Moreover, MSC-EVs enhanced PI3K–AKT activation in ovarian tissue, subsequently promoting angiogenesis and nutrition supply via the elevation of VEGF expression, thereby aiding follicular development and luteinisation. The findings, in conjunction with information from Castaño, Núñez López, Fang, and Jiang, substantiate a compelling argument for multi-modal EV-based therapies that simultaneously address systemic metabolic dysregulation and localised ovarian disease [176,232,233,235].

The integration of EV-based therapies in the treatment of PCOS requires a precision medicine approach. Through the application of EV cargo profiling, physicians may identify patient-specific molecular signatures, such as diminished miR-320a (associated with poor ART results) or elevated miR-21/miR-155 (related to chronic inflammation) [236]. This would enable the formulation of personalised medications. Potential strategies include inhibiting the release of detrimental EVs via anti-inflammatory diets, pharmacological inhibition of ceramide synthesis, or administering metformin to normalise EV miRNA content; alternatively, one may utilise engineered EVs enriched with insulin-sensitizing or anti-apoptotic cargo for ovarian delivery. The primary therapeutic aim is to re-establish a physiologically balanced EV network that harmonises metabolic, inflammatory, and reproductive pathways, thereby enhancing systemic health, restoring ovulatory function, and improving fertility outcomes in obese patients with PCOS, especially those receiving ART.

The translational significance of these findings is that they enable researchers to associate distinct extracellular vesicle cargo fingerprints with functional outcomes, hence facilitating the development of precision medicine strategies. The integration of omics-based extracellular vesicle profiling with clinical phenotyping enables the identification of pathogenic extracellular vesicle patterns specific to individual patients, facilitating the development of tailored therapies, including lifestyle modifications or the utilisation of extracellular vesicle-based medication delivery systems. Moreover, the alignment of preclinical evidence (e.g., BAT-Exos reinstating ovarian function through STAT3 inhibition and GPX4 activation) with human observational studies (e.g., miR-320a forecasting ART success) highlights the feasibility of advancing EV-based diagnostics and therapeutics from the laboratory to clinical practice. This synthesis demonstrates that the abnormal EV signalling axis in obese PCOS functions as both a catalyst and a marker of the illness state, providing a thorough molecular-clinical framework for clarifying its complex pathogenesis. This foundation smoothly transitions into the next discussion on potential intervention options, including the modification, supplementation, or targeting of EVs within the context of ART and metabolic restoration.

### 5.3. EVs as Diagnostic and Prognostic Biomarkers in Obese PCOS

A growing body of translational research increasingly identifies extracellular vesicles, particularly small extracellular vesicles, as specialised, cell-origin-specific messengers whose cargo can be utilised for diagnostic and prognostic purposes in obese polycystic ovary syndrome [237]. The molecular structure of these vesicles reflects the combined effects of metabolic disruptions, hyperandrogenism, chronic inflammation, and dysfunctional follicular signalling. The obese PCOS phenotype is characterised by the interplay of adipose tissue inflammation, insulin resistance, and modified steroidogenesis, resulting in the reconfiguration of EV biogenesis pathways, particularly those dependent on ESCRT machinery and ceramide-mediated budding. These alterations result in the increased concentration of specific regulatory miRNAs, piRNAs, proteins, and lipids in EVs, so modifying the cargo profile in a manner that significantly influences intercellular communication. This biassed molecular packaging disrupts communication among granulosa cells, theca cells, and the oocyte inside the follicular niche, thereby exacerbating maladaptive feedback loops in the PI3K–AKT–mTOR, MAPK–ERK, TGF-β–SMAD, and Wnt/β-catenin pathways [238]. Beyond the ovarian compartment, circulating extracellular vesicles function as systemic vectors, transmitting pathogenic signals to the hypothalamic-pituitary-ovarian axis, the endometrium, and metabolic organs, hence augmenting their potential as biomarkers for real-time systemic surveillance in obese polycystic ovary syndrome.

Muraoka and colleagues provided substantial clinical evidence by examining miRNAs and piRNAs in FF-derived sEVs from individual follicles of ART patients, identifying a distinctive three-miRNA signature—miR-16-2-3p, miR-378a-3p, and miR-483-5p—that reliably predicted clinical pregnancy [239]. These short RNAs are intricately involved in pathways essential for oocyte competency. miR-16-2-3p regulates CCND1 and CDK6, influencing the development and luteinisation of granulosa cells; miR-378a-3p impacts mitochondrial metabolism and angiogenesis via PGC-1α and VEGFA signalling; and miR-483-5p interacts with IGF2-related transcripts to modulate steroidogenesis [239]. The concentrations of these miRNAs varied between pregnant and non-pregnant follicles within the same patient, indicating intra-ovarian variability in EV-mediated signalling. The heterogeneity is likely more pronounced in obese PCOS, when insulin resistance, dyslipidaemia, and oxidative stress impose distinct stresses on particular follicles related to their milieu [197]. These findings suggest the possible use of EV-based signatures for follicle-specific prognostication, improving oocyte selection strategies in IVF.

Cui and colleagues explored further molecular details, demonstrating that FF-sEV miR-34a-5p is markedly elevated in PCOS and directly inhibits LDHA, thereby reducing the conversion of pyruvate to lactate [240]. This disturbance impedes anaerobic glycolysis in granulosa cells, reducing ATP availability during oocyte maturation. Concurrently, miR-34a-5p induces mitochondrial dysfunction, leading to the release of cytochrome c and the activation of caspase-3, so initiating apoptosis [240]. The decrease of AMPK activity and the resultant suppression of mTORC1-mediated protein synthesis worsen these metabolic and apoptotic abnormalities, both of which are essential for cytoplasmic maturation [241]. Inhibition of miR-34a-5p has been demonstrated to restore glycolysis in GC, halt apoptotic signalling, and normalise FSHR expression. This indicates that the restoration of metabolism is associated with increased follicular responsiveness [240]. In obese PCOS, where mitochondrial oxidative phosphorylation in granulosa cells is already impaired due to lipotoxicity, such therapies may produce greater advantages.

Liao and colleagues improved this understanding via integrative bioinformatics, outlining a network of 12 central miRNAs in FF-sEVs whose targets align with differentially expressed genes in PCOS oocytes [212]. Upregulated miRNAs, such as miR-93-3p and miR-152-3p, suppress PTEN and TSC1, leading to the dysregulated activation of PI3K–AKT–mTOR signalling and impaired autophagy [242]. In contrast, downregulated miRNAs, including miR-625-5p and miR-17-5p, impair the efficacy of the MAPK-mediated spindle assembly checkpoint. These alterations disrupt cell cycle regulation and cytoskeletal structure, increasing the likelihood of chromosomal misalignment, aneuploidy, and diminishing the possibility for blastocyst development in oocytes. In obese PCOS, these effects are intensified by the combined impact of hyperinsulinemia, which enhances AKT phosphorylation and hastens meiotic mistakes.

Duval and associates amalgamated multi-cohort datasets and identified a persistent dysregulation of miR-379 and constituents of the miR-200 family in the FF-EVs of PCOS [135]. These miRNAs modulate TGF-β–SMAD and Wnt/β-catenin signalling pathways, which are crucial for GC proliferation, extracellular matrix remodelling, and cumulus expansion. In obesity-related PCOS, heightened leptin levels and increased circulating TNF-α further alter EV cargo composition towards pro-inflammatory and anti-differentiation patterns, worsening oocyte maturation abnormalities [243]. The absence of generally conserved miRNA biomarkers across investigations underscores the challenges posed by technical variability in EV isolation, quantification, and normalisation, emphasising the need for rigorous standardisation before therapeutic implementation.

Esfandyari and colleagues introduced a non-miRNA biomarker perspective by demonstrating elevated levels of EV-associated DENND1A.V2 RNA and S100-A9 protein in patients with PCOS [244]. The overexpression of DENND1A.V2 enhances androgen production via CYP17A1, whereas S100-A9 functions as a TLR4 ligand, initiating the release of NF-κB-dependent pro-inflammatory cytokines in granulosa cells and theca cells [245]. In obese PCOS, adipose-derived EVs enriched with resistin, leptin, and palmitate-containing phospholipids aggravate insulin signalling deficiencies in GCs by modifying IRS1/2 phosphorylation. This establishes a pathogenic cycle that sustains hyperandrogenism and inflammation [246].

The findings together endorses a paradigm in which EVs serve as dynamic molecular sentinels in obese PCOS, proficient in recording both the local follicular milieu and the systemic metabolic condition. Their cargo signatures not only indicate potential for early detection of subclinical follicular and metabolic issues, but also provide insights into ART outcomes, including fertilisation efficacy, embryo development, implantation likelihood, and chances of live birth. The integration of EV-derived miRNA panels (miR-16-2-3p, miR-378a-3p, miR-483-5p, miR-34a-5p, miR-93-3p, and miR-152-3p) with protein markers (DENND1A.V2 and S100-A9), alongside lipidomic profiling and AI-assisted analysis, may facilitate precision reproductive medicine tailored to the molecular phenotype of each obese PCOS patient.

Table 3 summarising significant EV cargo molecules identified in the follicular fluid and blood of women with obese PCOS, their molecular targets, dysfunctional signalling pathways, and the clinical outcomes associated with ART. Recent experimental and clinical studies have compiled data on microRNAs, proteins, and lipids, along with functional annotations and mechanistic implications for follicular physiology and systemic metabolic regulation.

Table 3 consolidates current molecular data on EV cargo alterations in obese PCOS, highlighting their various roles in regulating granulosa cell metabolism, oocyte maturation, and endocrine-metabolic connections. Dysregulated microRNAs located within extracellular vesicles, including miR-16-2-3p, miR-378a-3p, miR-483-5p, miR-34a-5p, miR-93-3p, and miR-152-3p, influence pathways such as PI3K–AKT–mTOR, MAPK–ERK, AMPK, and TGF-β–SMAD. This disrupts glycolysis, mitochondrial activity, cell cycle progression, and intercellular communication between cumulus and oocyte cells. Protein cargos such as DENND1A.V2 and S100-A9 enhance androgen synthesis and inflammatory signalling, whilst alterations in the lipid content of adipose-derived extracellular vesicles exacerbate insulin resistance and lipotoxic stress. These vesicular signals may serve as early non-invasive biomarkers for reproductive prognosis and as therapeutic targets for restoring follicular competency and improving ART success rates in obese PCOS.

### 5.4. Limitations, Challenges, and Regulatory Considerations

Despite the innovative potential of EV-based diagnostics and treatments for treating obese PCOS, their practical application is hindered by a combination of technical, biological, and regulatory obstacles. The variability in EV cargo composition is a significant restriction at the molecular level. EVs derived from adipose tissue, granulosa cells, or immune cells can display significant variability in their miRNA, circRNA, lncRNA, protein, and lipid profiles, even when originating from the same patient, due to differences in metabolic state, hormonal environment, and systemic inflammation. These alterations affect the quantity of cargo molecules that regulate critical pathways such as PI3K–AKT–mTOR, AMPK–ACC, MAPK–ERK, and TGF-β–SMAD. These processes are crucial for glucose homeostasis, lipid metabolism, follicular development, and granulosa cell steroidogenesis. In obese PCOS, the altered inflammatory milieu—driven by TNF-α, IL-6, and resistin—can induce the release of EVs enriched with pro-inflammatory microRNAs that disrupt IRS phosphorylation, impede GLUT4 translocation, and modify FOXO1-mediated transcription, thereby exacerbating insulin resistance.

A major methodological challenge is the lack of consistency in techniques for extracellular vesicle separation and characterisation. Differential ultracentrifugation, size-exclusion chromatography, precipitation-based kits, and microfluidic devices yield extracellular vesicle populations distinguished by diverse size distributions, surface marker densities, and cargo compositions. Methodological disparities directly affect biomarker repeatability; for instance, an EV-derived miR-103 signal correlated with BMI in obese PCOS may be dependably detected by SEC but may be diminished or lost when utilising precipitation-based approaches. Similarly, quantifying the quantity of EVs using NTA may not correlate with protein assays such as the BCA method, complicating the comparison of research. In the absence of consensus on the minimal characterisation requirements, encompassing particle size (utilising NTA/TEM), surface markers (CD9, CD63, CD81), and cargo profiling, it remains challenging to identify clinically reliable EV-based biomarkers.

The targeted administration of synthetic extracellular vesicles in therapy presents both biological and technological problems. For example, including FSHR-targeting ligands onto EV membranes could potentially facilitate their uptake by granulosa cells; however, this may be less effective due to the variability of receptors at different follicular phases and among various forms of PCOS. Moreover, EV biodistribution investigations have revealed considerable off-target accumulation in the liver, spleen, and lungs, where macrophage absorption via scavenger receptors may lead to the lysosomal destruction of therapeutic cargo. This raises concerns over the efficacy of the treatment and the inadvertent stimulation of NF-κB, JAK–STAT, or p38 MAPK signalling in tissues outside the ovaries, potentially leading to systemic inflammatory or immunomodulatory consequences. In obese PCOS, modified lipid metabolism might alter the composition of extracellular vesicle membranes (for instance, by increasing cholesterol and sphingomyelin), impacting both their stability and their fusion efficiency with target cells.

The intracellular fate of externally introduced EVs is a mostly unexplored area. Target cells internalise EVs by clathrin-mediated endocytosis, caveolin-dependent entrance, or micropinocytosis, allowing for their sequestration in endosomes and subsequent delivery to lysosomes for degradation. This renders their cargo less accessible. Strategies that bypass endo-lysosomal trafficking, such as membrane fusion via viral glycoprotein integration or fusogenic lipids, are yet experimental and carry the potential for off-target effects. Furthermore, EV cargo, particularly miRNAs and transcription-modulating proteins, might induce epigenetic reprogramming in target tissues. This may facilitate the restoration of normal folliculogenesis; however, it also presents safety concerns if chromatin remodelling in oocytes or granulosa cells is compromised, potentially leading to long-term repercussions on embryonic development and offspring health.

From a regulatory and ethical perspective, EV-based therapies occupy an ambiguous position between biologics and advanced therapy medical products (ATMPs). Various agencies, such as the FDA and EMA, categorise them individually. This ambiguity impacts efforts for intellectual property, clinical trial design, and adherence to manufacturing standards. To obtain regulatory approval, adherence to ICH-GCP and GMP standards is required. This necessitates the ability to replicate batches, maintain sterility, and precisely characterise both the EV source and the cargo. Ethical considerations are crucial in reproductive medicine, as therapies can influence not only maternal health but also the epigenetic and developmental programming of subsequent generations. Regulatory bodies may therefore necessitate multi-generational safety data, especially if EV cargo affects methylation patterns in genes that regulate embryonic development, cardiometabolic risk, or reproductive capacity.

To tackle these obstacles, the field needs advance towards multi-center EV reference biobanks that collect standardised, longitudinal samples from obese PCOS and control populations, enabling comprehensive validation of molecular markers. Advanced multi-omics pipelines integrating proteomics, transcriptomics, and lipidomics at the single extracellular vesicle level may reveal persistent cargo markers that are less influenced by technological variations. Furthermore, the incorporation of systems biology approaches to model EV–target cell interactions may enhance the selection of ideal treatment targets and the forecasting of systemic effects before clinical trials. Only via the integration of molecular standardisation, mechanistic validation, and regulatory foresight can EV-based diagnostics and treatments transition from experimental tools to dependable solutions for those with PCOS who are overweight.

## 6. Conclusions and Future Directions

A thorough synthesis of the current evidence indicates that EVs, particularly small sEVs, function as crucial molecular messengers that modulate the relationships between metabolic dysregulation, chronic inflammation, and reproductive dysfunction in obese PCOS. The cargo’s composition, comprising miRNAs, piRNAs, lncRNAs, proteins, and lipids, is not arbitrary; it results from the pathogenic reprogramming of donor cells induced by insulin resistance, hyperandrogenism, oxidative stress, and low-grade inflammation. These vesicles enable accurate communication among granulosa cells, theca cells, and the oocyte within the ovarian follicle, influencing essential molecular pathways such as PI3K–AKT–mTOR, MAPK–ERK, TGF-β–SMAD, Wnt/β-catenin, and STAT3–GPX4. This disrupted signalling environment complicates the growth of GCs, impairs mitochondrial metabolism, hinders cumulus expansion, and affects spindle assembly checkpoints, resulting in diminished oocyte competence. Circulating EVs function as systemic endocrine carriers, transmitting pathophysiological signals to the HPO axis, endometrium, and peripheral metabolic organs, thereby strengthening the metabolic–reproductive feedback loop linked to obese PCOS.

The simultaneous diagnostic and therapeutic functions of extracellular vesicles are significant from a translational perspective. MicroRNA profiles obtained from follicular fluid-derived small extracellular vesicles, including miR-16-2-3p, miR-378a-3p, miR-483-5p, miR-34a-5p, miR-93-3p, and miR-152-3p, alongside protein indicators such as DENND1A.V2 and S100-A9, have been identified as potential predictive biomarkers for assisted reproductive technology outcomes. Incorporating these molecular fingerprints into multi-omics systems that integrate transcriptomic, proteomic, and lipidomic data could enable the development of prediction models to assess follicle responsiveness, embryo viability, and implantation success. AI-driven analytics could enhance these predictions by including clinical, metabolic, and genetic aspects. This would facilitate the development of individualised ovulation induction procedures and embryo selection strategies. The concept of engineering EVs to deliver corrective molecular payloads—such as miRNA antagonists to mitigate glycolytic blockade in germ cells, or antioxidant enzymes to restore redox homeostasis—represents a groundbreaking development in targeted reproductive medicine.

Despite the potential of this research domain, several difficulties must be addressed before EV-based methodologies may be implemented in clinical environments for obese PCOS patients. Initially, it is essential to standardise the techniques for isolating, purifying, and characterising EVs to ensure consistent biomarker identification across various locations. This involves consensus on metrics for particle quantification (NTA, TRPS), surface marker panels (CD9, CD63, CD81, TSG101), and standards for cargo profiling. Secondly, comprehensive multicenter validation studies are necessary to ascertain the reliability of candidate EV signatures in ethnically and phenotypically heterogeneous obese PCOS groups, ideally within prospective ART cycles. Third, it is essential to conduct functional validation of these putative cargos both in vitro and in vivo to determine causal links between modifications in EV-mediated signalling and reproductive characteristics. Fourth, enhancements to therapeutic EV delivery platforms are necessary to augment ovarian tropism, achievable by modifying the ligands for FSHR, LHR, or integrin subunits. Simultaneously, they ought to minimise off-target biodistribution and macrophage-mediated clearance. Comprehensive long-term safety studies, including transgenerational analyses, are crucial for assessing the potential epigenetic and developmental effects of EV-mediated interventions on offspring health, particularly regarding the influence of EV cargos on chromatin remodelling and gene imprinting.

In the future, diagnostics and treatments utilising EVs may transform the treatment of obese PCOS from a reactive, symptom-focused methodology to a precision-guided strategy informed by molecular profiling and tailored intervention. By integrating standardised EV biomarker testing into ART procedures, doctors might classify patients according to their expected ovarian responsiveness and metabolic resilience, thus enabling more personalised and perhaps less invasive treatment approaches. The progress of bioengineered extracellular vesicle therapeutics may reveal novel approaches for addressing metabolic and reproductive disorders at the cellular level, potentially facilitating the restoration of follicular competence before irreversible ovarian damage occurs. Successful implementation of these novel concepts necessitates collaboration among genetic biologists, reproductive endocrinologists, bioengineers, and regulatory agencies. This will ensure that scientific advancement is accompanied by rigorous methodologies and ethical safeguards. If effective, EV-based solutions could revolutionise the therapy landscape for obese PCOS, increasing reproductive outcomes and enhancing the overall metabolic health of affected women, hence safeguarding the health of future generations.

## Figures and Tables

**Table 1:** EV-mediated ovary–periphery crosstalk in obese PCOS: Molecular cargo, target tissues, and pathophysiological consequences.

Source Tissue	EV Cargo	Target Tissue(s)	Molecular Pathways Activated	Pathophysiological Outcome
Ovary (Granulosa/Theca Cells)	miR-103, miR-125b, miR-21, HMGB1, oxidized phospholipids, lncRNAs, circRNAs	Adipose tissue, skeletal muscle, liver, endothelium, immune system	PI3K–AKT–mTOR inhibition, PPARγ suppression, NF-κB activation	Insulin resistance, lipolysis, FFA release, systemic inflammation
Adipose Tissue (Visceral)	miR-27a, miR-34a, ceramides, inflammatory lipids	Ovary (granulosa cells, theca cells)	Aromatase inhibition, mitochondrial dysfunction (MFN1/2↓, OPA1↓)	Reduced estrogen synthesis, hyperandrogenism
Skeletal Muscle	miR-29a, miR-34a, PP2A subunits, myomiRs (miR-1, miR-206)	Ovary (cumulus–oocyte complex), systemic circulation	AMPK–PGC1α suppression, ULK1 inhibition, ROS accumulation	Mitochondrial dysfunction in oocytes, impaired ATP production
Liver (Hepatocytes)	miR-122, miR-192, PTP1B, apolipoprotein fragments	Ovary, adipose tissue, systemic metabolism	PERK–eIF2α–ATF4 activation, LXR–SREBP1c lipogenesis	Increased gluconeogenesis, dyslipidemia, androgen excess
Endothelium	miR-155, miR-21, ICAM-1, VCAM-1 mRNA	Ovary (stroma, vasculature)	NF-κB–p65 activation, eNOS downregulation, endothelin-1 upregulation	Reduced ovarian perfusion, aberrant angiogenesis
Immune Cells (Macrophages/Monocytes)	S100A8/A9, oxidized cardiolipin, pro-inflammatory cytokine mRNAs	Ovary (granulosa cells), liver, skeletal muscle	NLRP3 inflammasome activation, MAPK p38/JNK signaling	Cytokine-induced androgen production, systemic insulin resistance

**Table 2:** EV-based diagnostics & therapeutics in obese PCOS—Strategies, targets, and translational readiness.

Category	EV Source	Cargo/Marker	Primary Target Tissue	Pathway Readout	Intended Outcome	Evidence Level	Translational Notes
Diagnostic	Plasma (circulating)	miR-122, miR-27b, miR-21, miR-103, miR-222, miR-320	Systemic (biomarker)	SREBP1/FASN; PI3K–AKT–mTOR; MAPK/ERK	Early detection of metabolic–reproductive imbalance	Clinical observational (pilot–moderate)	ddPCR/NGS panels; pre-analytical standardization required
Diagnostic	Serum (exosomes)	Proteins: p-AKT, SOCS3, adiponectin receptor fragments	Systemic (biomarker)	Insulin signaling integrity; IR severity	Risk stratification for IR-dominant phenotype	Preclinical + small clinical	Immunoassays; multiplex proteomics feasible
Diagnostic	Follicular fluid	miR-21, miR-93, miR-146a, miR-320; lncRNA H19; EV tetraspanins (CD63)	Ovary (local biomarker)	FSHR–cAMP–PKA; NF-κB; mitochondrial biogenesis	Oocyte competence and ART outcome prediction	Preclinical + IVF cohort correlative	In-theatre sampling; integrates with FF metabolomics
Diagnostic	Urine (non-invasive)	miR-21, miR-125b (renal-stable)	Systemic (biomarker)	Inflammation/HA surrogate	Screening adjunct for obese PCOS	Exploratory	Standardized isolation (SEC/TFF) advised
Therapeutic (Engineered EV)	Hepatocyte-derived EV	anti–miR-122 (antagomir)	Liver → Ovary (indirect)	SREBP1c; VLDL output; systemic insulin load	Reduce hepatic lipogenesis; alleviate ovarian HA	Preclinical (rodent)	Assess off-target lipid handling; biodistribution mapping
Therapeutic (Engineered EV)	Adipocyte-derived EV	miR-223 mimic	Ovary (TC/GC)	NF-κB dampening; IRS/PI3K balance	Lower ovarian inflammation; improve insulin sensitivity	Preclinical (in vitro/in vivo)	FSHR/LHR ligand decoration to enhance targeting
Therapeutic (Engineered EV)	Granulosa cell-derived EV	let-7 family mimics	Theca cells	IRS2–PI3K restraint; steroidogenesis normalization	Reduce androgen biosynthesis; restore E2/T balance	Preclinical (cellular)	Local intra-ovarian delivery during ART
Therapeutic (Engineered EV)	Generic producer line (HEK/MSC)	miR-320 mimic	Ovary (GC/CC)	MAPK–ERK–c-Fos modulation; proliferation/apoptosis	Enhance GC survival; support folliculogenesis	Preclinical (rodent follicles)	Potency release: qPCR + functional bioassay
Therapeutic (MSC-EV)	Mesenchymal stromal cells	SIRT1 protein; catalase/SOD2; VEGF-A/ANGPT1	Ovary (stroma/GC)	AMPK–PGC1α–NRF1; PI3K–AKT–eNOS	Mitochondrial rescue; anti-OS; pro-angiogenesis	Preclinical (rodent PCOS models)	GMP sourcing; immunogenicity and release criteria
Therapeutic (Inflammation)	Engineered EV	NLRP3 inhibitor (small molecule) or siNLRP3	Ovary (GC/immune)	Inflammasome inhibition; IL-1β/IL-18 reduction	Preserve follicular viability; reduce pyroptosis	Preclinical (in vivo)	Pyrogen testing; cytokine panel pharmacodynamics
Therapeutic (Metabolic)	Engineered EV	AMPK activator (e.g., AICAR derivative)	Ovary/Adipose/Skeletal muscle	AMPK–ULK1; GLUT4 trafficking; β-oxidation	Systemic insulin sensitization; reduce HA indirectly	Preclinical (multi-organ)	Targeting by tissue-specific peptides
System Modulation	Host (all tissues)	nSMase2 inhibition (e.g., GW4869)	Systemic (EV biogenesis)	Ceramide-dependent exosome release	Lower pathogenic EV burden	Preclinical	Monitor physiological EV functions; dosing windows
System Modulation	Host (adipose)	Ceramide synthase blockade; myriocin	Adipose → Ovary (indirect)	Sphingolipid remodeling; TLR4 tone	Reduce inflammatory EV cargo/uptake	Preclinical	Lipidomics-guided response monitoring
System Modulation	Host circulation	Decoy nanoparticles/EV traps	Systemic (sink)	Competitive binding to integrins/tetraspanins	Sequester pathogenic EV before uptake	Exploratory	Biodistribution and clearance profiling needed
Companion Dx	Plasma/FF	Multi-omic EV panel (miRNA + protein + lipid)	Systemic (biomarker)	IR/HA/Inflammation classifiers	Phenotype stratification; therapy selection	Preclinical + pilot clinical	ML models; cross-lab standardization

This table enumerates the current and novel methodologies by which EV-based assessments and therapies can assist individuals with PCOS who are overweight. It discusses the EV source, molecular payload, primary target tissue, significant pathway readouts, anticipated clinical results, evidence level, and translational considerations. Circulating serum, follicular fluid, and urine extracellular vesicle biomarkers are utilised for diagnosis, while manufactured extracellular vesicles targeting metabolic–reproductive axis malfunction are employed for treatment.

**Table 3:** Molecular signatures of extracellular vesicles in obese PCOS: Diagnostic, prognostic, and therapeutic perspectives.

EV Source	Key Biomarker/Cargo	Primary Molecular Targets	Involved Pathways	Clinical Relevance in Obese PCOS
Follicular fluid small EVs	miR-16-2-3p, miR-378a-3p, miR-483-5p	CCND1, CDK6, PGC-1α, VEGFA, IGF2-related transcripts	PI3K–AKT–mTOR, mitochondrial metabolism, steroidogenesis	Predicts clinical pregnancy; identifies high-quality oocytes in ART
Follicular fluid small EVs	miR-34a-5p	LDHA, AMPK–mTORC1 axis, cytochrome c, caspase-3	Glycolysis, oxidative phosphorylation, apoptosis regulation	Restores GC metabolism and viability when inhibited; potential therapeutic target
Follicular fluid small EVs	miR-93-3p, miR-152-3p, miR-625-5p, miR-17-5p	PTEN, TSC1, spindle assembly checkpoint components	PI3K–AKT–mTOR, MAPK, autophagy, chromosome segregation	Biomarkers of meiotic error risk and embryo developmental competence
Follicular fluid EVs	miR-379, miR-200 family	TGF-β–SMAD, Wnt/β-catenin pathway regulators	Follicular cell proliferation, ECM remodeling, cumulus expansion	Potential indicators of GC dysfunction and impaired folliculogenesis
Follicular fluid EVs	DENND1A.V2 RNA, S100-A9 protein	CYP17A1 (via DENND1A.V2), TLR4 (via S100-A9)	Steroidogenesis, NF-κB-mediated inflammation	Markers of hyperandrogenism and chronic ovarian inflammation
Serum/circulating EVs	miR-320a	HPO axis regulators, insulin signaling mediators	HPO axis signaling, reproductive endocrine feedback	Correlates with pregnancy outcomes in IR-positive PCOS
Adipose tissue-derived EVs	Resistin, leptin, palmitate-enriched phospholipids	IRS1/2 phosphorylation sites, inflammatory lipid metabolism enzymes	Insulin signaling disruption, NF-κB activation, lipotoxic stress	Mediators of systemic metabolic dysfunction impacting ovarian response

## Data Availability

No new data were created or analyzed in this study. Data sharing is not applicable to this article.
